# Completeness of HIV-1 Envelope Glycan Shield at Transmission Determines Neutralization Breadth

**DOI:** 10.1016/j.celrep.2018.09.087

**Published:** 2018-10-23

**Authors:** Kshitij Wagh, Edward F. Kreider, Yingying Li, Hannah J. Barbian, Gerald H. Learn, Elena Giorgi, Peter T. Hraber, Timothy G. Decker, Andrew G. Smith, Marcos V. Gondim, Lindsey Gillis, Jamie Wandzilak, Gwo-Yu Chuang, Reda Rawi, Fangping Cai, Pierre Pellegrino, Ian Williams, Julie Overbaugh, Feng Gao, Peter D. Kwong, Barton F. Haynes, George M. Shaw, Persephone Borrow, Michael S. Seaman, Beatrice H. Hahn, Bette Korber

**Affiliations:** 1 Theoretical Biology & Biophysics, Los Alamos National Laboratory, Los Alamos, NM 87545, USA; 2 Perelman School of Medicine, University of Pennsylvania, Philadelphia, PA 19104, USA; 3 Center for Virology and Vaccine Research, Beth Israel Deaconess Medical Center, Harvard Medical School, Boston, MA 02115, USA; 4 Vaccine Research Center, NIH, Bethesda, MD 20892, USA; 5 Duke Human Vaccine Institute, Duke University School of Medicine, Durham, NC 27710, USA; 6 Center for Sexual Health & HIV Research, Mortimer Market Centre, London WC1E 6JB, UK; 7 Human Biology Division, Fred Hutchinson Cancer Research Center, Seattle, WA 98109, USA; 8 Nuffield Department of Clinical Medicine, University of Oxford, Oxford OX3 7FZ, UK; 10 Senior author; 11 Lead Contact

## Abstract

Densely arranged *N*-linked glycans shield the HIV-1 envelope (Env) trimer from antibody recognition. Strain-specific breaches in this shield (glycan holes) can be targets of vaccine-induced neutralizing antibodies that lack breadth. To understand the interplay between glycan holes and neutralization breadth in HIV-1 infection, we developed a sequence-and structure-based approach to identify glycan holes for individual Env sequences that are shielded in most M-group viruses. Applying this approach to 12 longitudinally followed individuals, we found that transmitted viruses with more intact glycan shields correlated with development of greater neutralization breadth. Within 2 years, glycan acquisition filled most glycan holes present at transmission, indicating escape from hole-targeting neutralizing antibodies. Glycan hole filling generally preceded the time to first detectable breadth, although time intervals varied across hosts. Thus, completely glycan-shielded viruses were associated with accelerated neutralization breadth development, suggesting that Env immunogens with intact glycan shields may be preferred components of AIDS vaccines.

## In Brief

Wagh et al. show that transmitted viruses with more intact glycan shields are correlated with development of neutralization breadth in HIV-1-infected individuals. This is consistent with previous findings that glycan holes in Env immunogens are targeted by strain-specific neutralizing responses, and suggests that immunogens with intact glycan Shields may be advantageous.

## Graphical Abstract

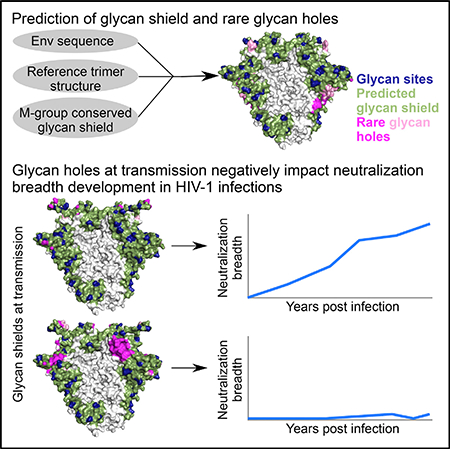

## INTRODUCTION

A characteristic feature of HIV type 1 (HIV-1) is the extensive glycosylation of its envelope (Env) glycoprotein. Glycans are added at potential N-linked glycosylation sites (PNGSs) as the Env protein traffics through the endoplasmic reticulum (ER) and Golgi network. Averaging 93 PNGSs per Env trimer, glycans comprise roughly half its mass (Behrens and Crispin, 2017) and shield ~70% of the protein surface from antibodies (Pancera et al., 2014). The number of PNGSs per gp120 subunit varies dramatically (18–33 PNGSs [Zhang et al., 2004]) and varies greatly even within a single host (Bonsignori et al., 2017; Liao et al., 2013; Wei et al., 2003). PNGSs also often shift; e.g., a common N332 to N334 shift results in resistance to V3-glycan antibodies (Freund et al., 2017; Moore et al., 2012). Glycans are highly dynamic (Lemmin et al., 2017; Stewart-Jones et al., 2016; Tian et al., 2016a; Yang et al., 2017), and a single PNGS can be occupied by different glycoforms due to glycan processing (Behrens et al., 2016; Cao et al., 2017; Go et al., 2017). As host proteins are also glycosylated by the same pathways, tolerance mechanisms often impede anti-glycan antibody development (Haynes and Verkoczy, 2014). These features render the HIV-1 Env “glycan shield” a formidable defense against antibody responses.

The earliest neutralizing antibodies (NAbs) following infection are specific for the transmitted founder (TF) virus and often select for escape mutations that alter the TF glycan shield (Bar et al., 2012; Bonsignori et al., 2017; Frost et al., 2005; Moore et al., 2009; Richman et al., 2003; Wei et al., 2003). After multiple rounds of immune selection and viral escape, some subjects develop NAbs that can neutralize most genetically divergent HIV-1 strains (Bhiman et al., 2015; Bonsignori et al., 2016; Doria-Rose et al., 2014; Gao et al., 2014; Hraber et al., 2014; Liao et al., 2013; MacLeod et al., 2016; Moore et al., 2012). Such broadly NAbs (bNAbs) often target a few common “sites of vulnerability” on the Env trimer: the CD4-binding site (CD4bs), a high mannose patch at the base of variable loop 3 (V3), the trimer apex, the gp120-gp41 interface, the fusion peptide, and the membrane-proximal external region (MPER) (Burton and Hangartner, 2016; Kwong et al., 2013). Each bNAb class interacts with both protein and glycans (Andrabi et al., 2015; Gorman et al., 2016; Lee et al., 2016; McLellan et al., 2011; Sok et al., 2014; Stewart-Jones et al., 2016).

BNAbs are associated with longer duration of infection, more effective CD4^+^ T cell help, high viral loads, and plasma autoantibodies (Cortez et al., 2012; Landais et al., 2016; Moody et al., 2016; Moore et al., 2015; Piantadosi et al., 2009; Rusert et al., 2016), and viral diversification often precedes bNAb development (Bhiman et al., 2015; Bonsignori et al., 2016; Doria-Rose et al., 2014; Gao et al., 2014; Liao et al., 2013; MacLeod et al., 2016; Moore et al., 2012). Also, certain Envs can bind the unmutated common ancestor (UCA) of bNAb lineages (Andrabi et al., 2015; Bhiman et al., 2015; Bonsignori et al., 2017; Gorman et al., 2016; Liao et al., 2013). Despite these insights, particular TF Env features that clearly predict bNAb development have not been identified. Relative NXT versus NXS PNGS motif abundance may play a role, but conflicting results have been reported (Smith et al., 2016; van den Kerkhof et al., 2013).

Env immunogens lacking certain PNGSs can elicit NAbs that preferentially target the unshielded regions in animal vaccination studies (Bradley et al., 2016; Crooks et al., 2015; Klasse et al., 2016; McCoy et al., 2016; Pauthner et al., 2017; Sanders et al., 2015; Torrents de la Peña et al., 2017; Zhou et al., 2017). When such breaches in the Env glycan shield (glycan holes) are rare (e.g., the loss of the PNGS at N241 in BG505 occurs in only ~5% of M-group Envs), it is not surprising that NAbs targeting them lack breadth. It is not clear whether NAbs targeting immunodominant glycan holes have the capacity to broaden, or whether they hinder development of subdominant Abs with greater potential to acquire breadth.

Here, we explored the role of glycan holes in the development of neutralization breadth during natural infection in a longitudinal study of 12 HIV-1-infected subjects by developing a sequence and structure-based computational approach to identify glycan holes and using this strategy to compare glycan shield evolution to bNAb development. The predicted Env glycan hole area at transmission was negatively correlated with the maximum neutralization breadth that developed years later. Most TF Env glycan holes were filled within 2 years of infection, at least in part due to escape from autologous glycan-hole targeting NAb responses. In individuals where the transmitted virus had sizeable glycan holes, the evolution to a more intact glycan shield generally preceded or coincided with the onset of heterologous breadth. Thus, viruses with a more intact Env glycan shield are associated with the development of broader neutralizing responses, suggesting that Env immunogens with a more complete glycan shield may be needed to elicit bNAbs.

## RESULTS

### Computational Prediction of Glycan Holes

While crystal and cryoelectron microscopy (cryo-EM) structures provide critical molecular details of the Env glycan shield (Gristick et al., 2016; Lee et al., 2016; Pancera et al., 2014; Stewart-Jones et al., 2016), they do not readily translate between variants and do not account for glycan dynamics. Molecular dynamics (MD) simulations have begun to address the latter but have not yet incorporated native glycans (Lemmin et al., 2017; Yang et al., 2017), and it is not clear how to extract from MD the features of the glycan shield that are relevant for Nab-Env interactions. Here, we developed a computational approach to visualize and quantify the extent of the surface area exposed by infrequent glycan holes for a given Env sequence.

**Figure 1.**
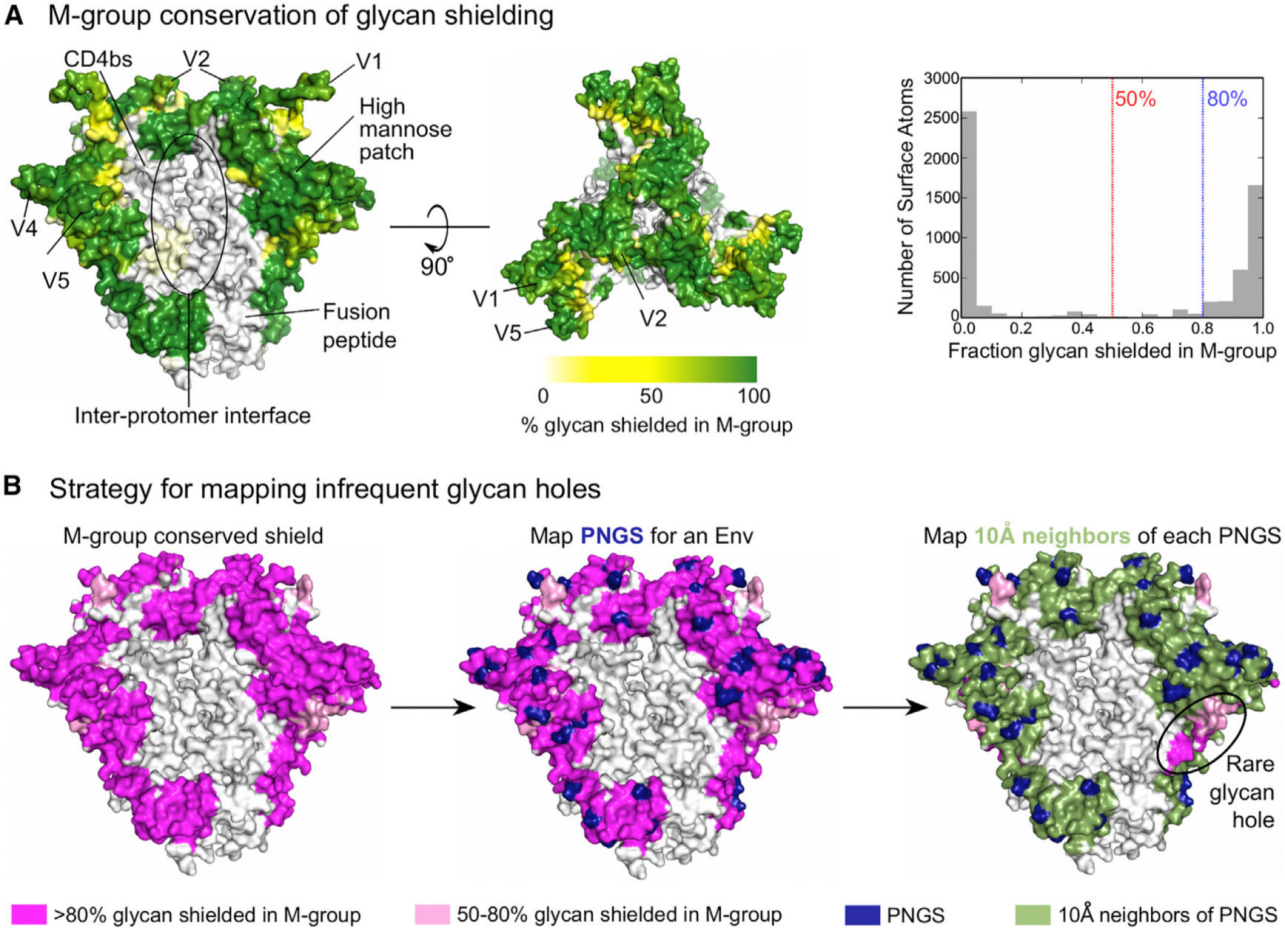
Conservation of the Glycan Shield in M-Group Viruses and Glycan Shield Mapping Strategy (A) The percentage of M-group Envs in which a given protein surface region is glycan shielded is depicted using a white-yellow-green color gradient. Env regions of interest are indicated. The white area labeled as the inter-protomer interface in the left panel is a cleft (Video S1) with low glycan coverage (Stewart-Jones et al., 2016). The right panel shows the distribution of glycan shield conservation in M-group Envs for surface-exposed atoms. (B)Glycan shield mapping strategy. The starting point is the Env trimer color coded according M-group glycan shield conservation with the <50%, 50%–80%, and >80% conserved regions by white, pink, and magenta, respectively (left). The PNGS positions (blue) for a given sequence are first mapped onto this structure (middle). Next, trimer surface regions within a 10-Å radius of each PNGS are mapped as green (right). This approach automatically highlights infrequent glycan holes.

We first map PNGSs for a given Env sequence onto a reference trimeric Env crystal structure (see STAR Methods), assuming each PNGS is glycan occupied. To approximate glycan dynamics, we assume that each glycan shields the Env protein surface within a 10-Å radius of the PNGS asparagine alpha carbon atom, a cutoff previously used to characterize the Env protein surface in the vicinity of glycans (Stewart-Jones et al., 2016). By mapping shielded areas around each PNGS onto the trimer protein surface, the extent of glycan shielding for an individual Env can be estimated (Figure 1). While not explicitly showing glycans, this visualization shows the protein surface predicted to be glycan shielded for a given Env variant.

To characterize glycan shield conservation among diverse M-group viruses, we applied this approach to 4,582 Env sequences from the Los Alamos HIV Database and calculated the frequency of shielding for every surface-exposed atom on the Env trimer (Figure 1A). Levels of glycan shield conservation were bimodally distributed (Figure 1A, right). Based on this distribution, we defined two cutoffs: atoms shielded in either >50% or >80% of M-group Envs. To map rare holes in the glycan shield of an Env of interest, we superimposed its predicted glycan shield coverage onto the protein surface that is typically covered based on these two cutoffs (Figure 1B). Data S1 provides a PDB file with M-group conservation of glycan shielding, and Video S1 is a 3D visualization of M-group glycan shield conservation on the Env trimer.

This approach confirmed that most of the outer domain of the Env trimer is glycan shielded across most diverse pandemic HIV-1 strains (Figure 1A). In contrast, recessed regions close to the inter-protomer axis, the CD4-binding site, and the fusion peptide were almost never shielded (Figure S1A). These regions not only lacked PNGSs, but also did not have any structurally proximal PNGSs in any of 4,582 M-group Envs examined (Figure S1F). This suggests evolutionary constraints to retain proper function, folding and/or trimerization, and is consistent with their accessibility to some bNAbs (Zhou et al., 2015) (Figures S1B and S1C). As these regions are always glycan free, they cannot, by definition, have infrequent glycan holes and were excluded from analyses.

Our approach accounts for compensatory shielding by neighboring PNGSs. For example, the high-mannose patch around N332 was predicted to be shielded in 98%–100% M-group Envs, even though particular PNGSs in this region, such as N332 and N295, are present in only 58%–71% M-group Envs (Figures S1D and S1E). It does not account for variation in glycan occupancy or heterogeneity of glycoforms and has a simplistic treatment of glycan dynamics. Predictions of glycan shielding within hypervariable regions are inherently problematic as they have high structural flexibility, and extreme sequence and length variation confound alignments in these regions. Thus, glycan-shield mapping results for hypervariable regions should be treated with caution; we showed the approximate shielding of these regions in figures but excluded them from the quantitative analyses below.

### Validation of the Glycan Shield Mapping Strategy

**Figure 2.**
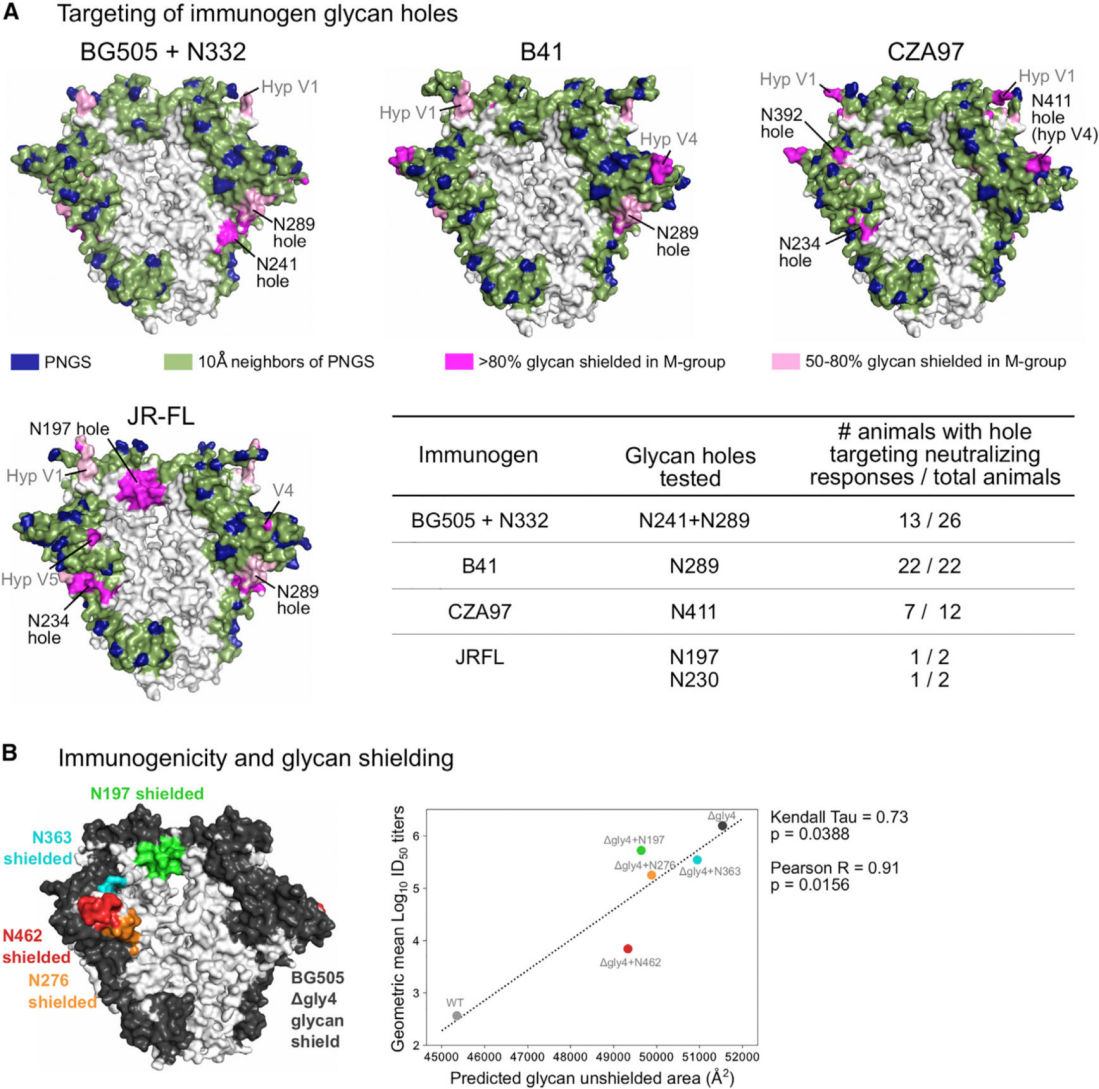
Validation of Glycan Shield Mapping Strategy (A) Predicted glycan shields for trimeric Env immunogens. PNGSs are indicated by dark blue and glycan shielded regions in green. Strain-specific breaches in the glycan shields are highlighted in magenta (regions shielded in >80% M-group Envs) or pink (shielded in 50%–80% M-group Envs). The table summarizes the results from epitope mapping of autologous NAb responses from Klasse et al. (2016) and Crooks et al. (2015), with the number of animals with reduced activity against glycan knockin mutants and the total number of animals with autologous neutralization activity reported. (B)Predicted glycan shielding (left) of the four PNGS-deleted BG505 variant BG505-Δgly4 from Zhou et al. (2017) is shown in black, and the alterations to the glycan shielded area due to the addition of each of the four deleted PNGSs are shown in green (PNGSs at N197 added), cyan (N363), red (N462), and orange (N276). The panel to the right shows the correlation between the glycan hole area and geometric mean Log_10_ ID_50_ neutralization titers against the indicated Envs using sera from BG505-Δgly4-immunized guinea pigs.

To test our ability to identify immunologically relevant glycan holes, we retrospectively analyzed four trimeric Env immunogens that had elicited glycan hole-targeting strain-specific NAb responses (Klasse et al., 2016; Crooks et al., 2015). In each case, we correctly predicted glycan holes in the experimentally identified immunodominant epitopes (Figure 2A). Klasse et al. noted the absence of 3 glycans in their BG505+N332 immunogen, one at N130 and two that are structurally adjacent, N241 and N289. Our method predicted a large hole encompassing N241 and N289, but not around N130, as the area around N130 was shielded by other neighboring glycans. Half of the 26 BG505+N332 SOSIP-vaccinated rabbits developed autologous NAb responses that could be blocked by glycan addition at either N241 or N289 or both. However, glycan addition at N130, where we did not predict a hole, did not impact neutralization capacity of the vaccine sera. Similarly, an N289-centered glycan hole predicted in B41 was targetedin22/22B41 SOSIP-immunized rabbits (Klasse et al., 2016). A third immunogen, CZA97, exhibited several glycan holes (Figure 2A), with only the N411 hole experimentally tested; it was targeted in 7/12 CZA97-vaccinated animals. Finally, Crooks et al. showed that JRFL-vaccinated animals that make potent type-specific neutralization responses targeted either the predicted N197 or the N234-N289 glycan holes (Figure 2A).

Our strategy also recapitulated a demonstrated quantitative relationship between the glycan hole area and vaccine-elicited NAb titers (Zhou et al., 2017). Zhou et al. designed immunogens with glycan holes (Δgly) around the CD4 binding site, such as BG505-Δgly4 with PNGS removal at positions 197, 276, 363, and 462. They immunized guinea pigs with BG505-Δgly4 SOSIP trimer and tested post-vaccination NAb responses against pseudoviruses with wild-type BG505 and BG505-Δgly4 Envs as well as with four mutant BG505-Δgly4 Envs, each with a single PNGS added back (e.g., BG505-Δgly4 + N197). Potent NAb responses targeting the glycan-exposed protein surface near the CD4 binding site were elicited, with very little cross-reactivity with the WT virus. The glycan hole area for each Env variant was estimated using MD simulations and was significantly correlated with vaccine-elicited neutralization ID50 titers. Calculating the glycan hole area with our computationally rapid algorithm confirmed the quantitative relationship (Figure 2B), albeit with lower accuracy than the computationally intensive MD simulations (using MD simulations, Zhou et al. found a Pearson R = 0.99, our method yields R = 0.91). Nonetheless, these analyses show that our method captures both qualitative and quantitative immunologically relevant aspects of the Env glycan shield.

### The Extent of the Glycan Shield at Transmission Correlates with Development of Heterologous Neutralization Breadth

**Figure 3.**
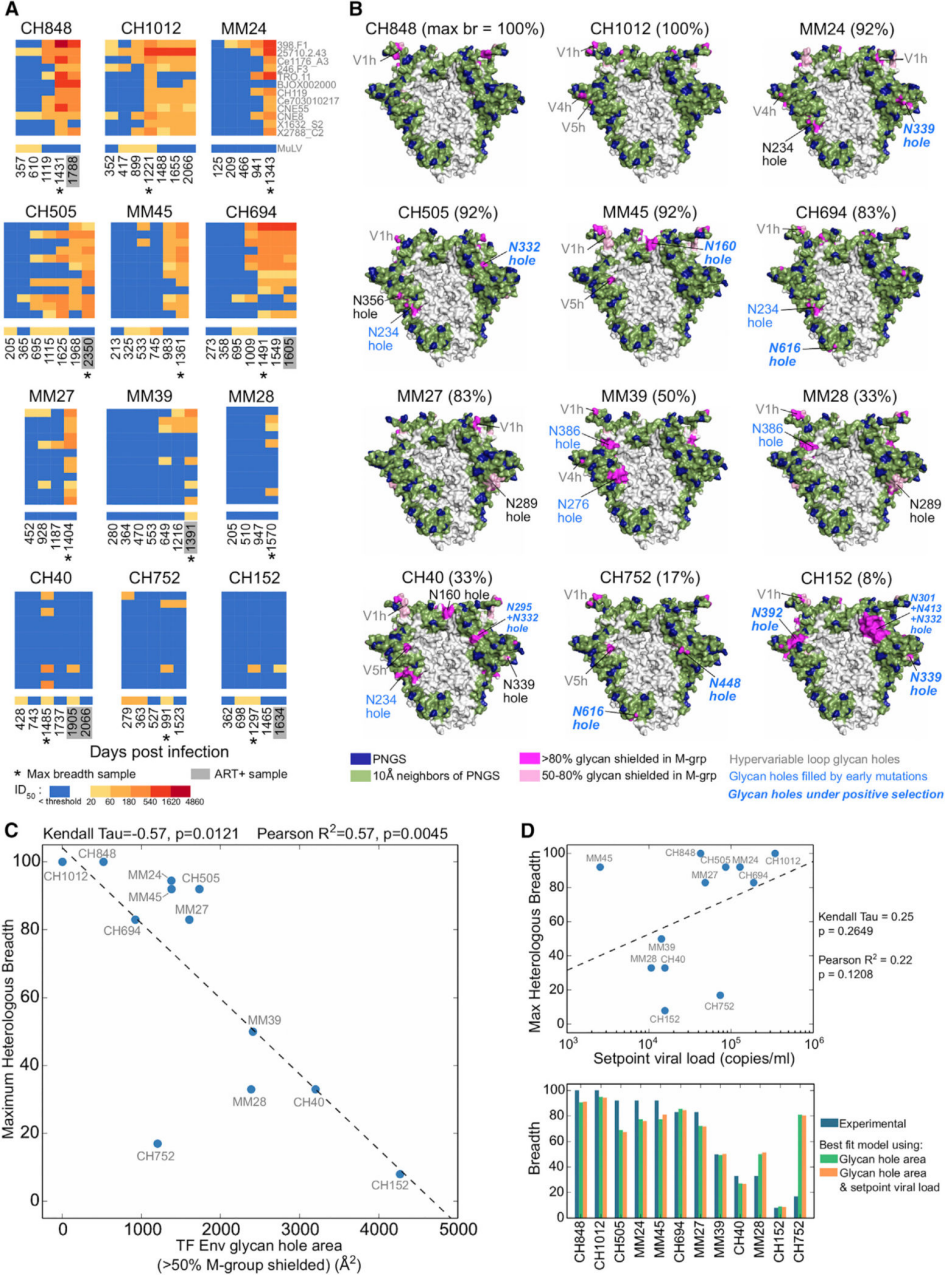
The Size of the TF Glycan Holes at Transmission Is Negatively Correlated with Maximum Heterologous Neutralization Breadth (A) Heatmaps of plasma ID50 neutralization titers against the 12-virus global panel (deCamp et al., 2014) color coded by potency. Lack of neutralization is shown in blue. MuLV ID50 titers are controls. Asterisks indicate the earliest time point of maximum breadth. Gray boxes indicate anti-retroviral therapy (ART) positive plasma samples, tested using ART-resistant pseudoviruses. See Table S3 for ID50 values. (B) Predicted glycan shields for the TF Envs of the 12 individuals studied. The percentage of maximum breadth developed for each individual is shown in brackets. Colors are as in Figure 1B. Absent PNGSs leading to glycan holes are indicated; those filled during infection are highlighted in blue (see Figures 4, 5, and 6). TF glycan holes with significant positive selection signatures (Table S5; Figure S6) are in bold italics. Glycan holes in hypervariable loops, which cannot be predicted reliably, are labeled gray. (C) Correlation between maximum heterologous plasma breadth and the TF glycan hole area, using the >50% M-group conserved glycan shield threshold (hypervariable loop glycan holes excluded). For MM24 and MM45, data points were overlapping and are separated along the y-axis for clarity. (D) Top: correlation between maximum plasma breadth and setpoint viral load. Bottom: comparison of maximum plasma breadth (blue bars) with best-fit values using a model with TF glycan hole area alone (green) and a model that includes both the TF glycan hole area and setpoint viral load (orange).

To explore the impact of Env glycan shields on neutralization breadth development during HIV-1 infection, we studied 12 subjects from transmission through 2.6–7.6 years postinfection (Table S1). Each subject was infected with a single TF virus, which could be readily inferred (Keele et al., 2008). We characterized the evolving Env quasispecies in these subjects using single genome amplification (SGA) to generate 132–1,244 full-length env sequences from 7–35 plasma samples per subject (Table S2). Phylogenetic trees showed most subjects had a typical pattern of evolutionary divergence from the TF, although three subjects, MM24, MM45, and CH40, showed relatively low diversification over time (Figures S2 and S3). For each subject, 4–7 plasma samples were tested for neutralization breadth against the standard global panel of 12 tier 2 heterologous pseudoviruses (deCamp et al., 2014). The 12 subjects developed a wide range of heterologous neutralization responses (Figure 3A; Table S3). At the extremes, 3- to 4-year plasma from subjects CH848 and CH1012 potently neutralized all 12 pseudoviruses, while at comparable time points, plasma from CH752 and CH152 neutralized only one or two. Among the 12 subjects, CH505, CH848, CH694, CH1012, MM24, MM27, and MM45 developed high breadth (maximum breadth = 83%–100%), and CH40, CH152, CH752, MM28, and MM39 developed low breadth (maximum breadth = 8%–50%).

To compare the extent of glycan shielding at transmission to subsequent neutralization breadth development, we predicted the glycan shields for each of the 12 TF Envs and calculated the total solvent-accessible surface area for their glycan holes (STAR Methods) (Figure 3B). There was considerable variability, ranging from near complete glycan shields (CH848 and CH1012 TFs) to the presence of multiple glycan holes (CH40, CH752, and CH152 TFs). The glycan hole area distribution for these 12 TF Envs was not unusual but was comparable to the distributions found across M-group Envs, subtype B TF Envs (Keele et al., 2008), and subtype C early/acute Envs (Rademeyer et al., 2016). In each of the 4 datasets, complete shields were most common (Figures S1G and S1H).

A significant negative correlation was found between the total glycan hole area at transmission and the maximum neutralization breadth that developed 3.6–7.6 years later (Figure 3C). This correlation was robust in that significance was maintained using both cutoffs for glycan shield conservation (>50%, p = 0.012, and >80%, p = 0.037, Kendall’s Tau rank test) (Figures 3C and S4A), as well as when using two alternative thresholds for neutralization activity (p = 0.0015–0.0085, Kendall’s Tau rank test; see STAR Methods; Figures S4B and S4C). We also explored the impact of using a different Env reference structure (subtype A BG505 trimer crystal structure; PDB: 5FYL) for calculation of infrequent glycan holes. The BG505 structure yielded a highly similar map of glycan shield conservation and reproduced the significant negative correlation between breadth and the infrequent TF glycan hole area (p = 0.005, Kendall’s tau rank test) (Figure S4D). Since using an alternate Env structure had no appreciable effect on the glycan shield prediction, we continued to use the X1193 reference structure for all subsequent calculations. Finally, we varied the assumed glycan shielding radius cutoff from 5 to 20 Å. The negative correlation between the TF glycan hole area and neutralization breadth was robust over a range of glycan shield radius cutoffs, with significant correlations (p < 0.05) found for 8- to 15-Å cutoffs (Figures S5A– S5C). Depending on the correlation statistic used, the best cutoffs were either 15 or 10 Å. Since the 10-Å cutoff most accurately predicted TF glycan holes that were targeted by autologous neutralizing responses (see below), we used this cutoff for all subsequent analyses.

We next tested two alternative approaches for glycan hole characterization. First, we used the entire Env surface, including regions that were rarely or never shielded in M-group Envs, to calculate the glycan hole area for each TF. A weaker association was found between the total TF glycan hole area and maximum breadth, relative to focusing just on breaches in the conserved glycan shields (Figure S4E). Second, using an alignment approach based on simply tallying the number of missing common PNGSs did not detect a significant association between the number of missing PNGSs and neutralization breadth (Figure S4F). Thus, the negative correlation between the TF glycan hole area and neutralization breadth required both structural and M-group conservation information.

Finally, we tested whether setpoint viral load (SVL), a known correlate of bNAb development (Moore et al., 2015; Rusert et al., 2016), impacted our results. In our data, there was no significant association between SVL and maximum breadth (Figure 3D, p = 0.2649, Kendall’s Tau), likely because our dataset is much smaller than previous cohorts that established this relationship (Rusert et al., 2016). We then used a generalized linear model that included SVL as an additional independent variable to re-examine the relationship of the TF Env glycan hole area and maximum breadth. While the TF Env glycan hole area significantly contributed to the model (p = 1.22 × 10^–7^), the addition of SVL to the model did not significantly improve its accuracy (Figure 3D; difference in Akaike information criterion [AIC] = 1.89). Thus, TF glycan hole area predicted plasma neutralization breadth both more strongly and independently of SVL.

### Glycan Holes in the CH152 TF Are Targeted by Autologous NAbs

**Figure 4.**
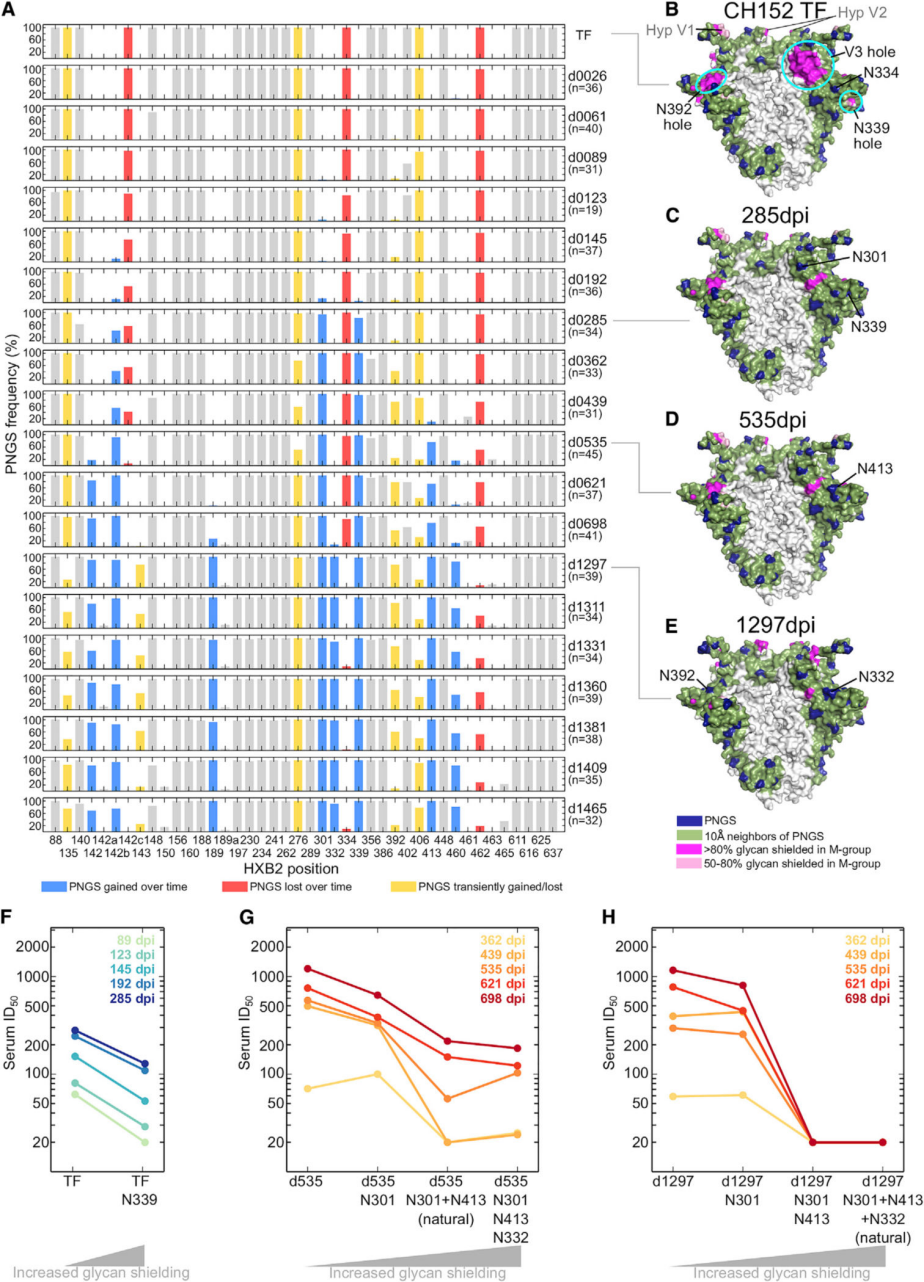
Evolution of the Env Glycan Shield in an Individual Who Failed to Develop Heterologous Neutralization Breadth (A) Losses and gains of PNGSs in CH152 longitudinal sequences. The frequencies of PNGSs within Env sequences from each time point are indicated; the time point (e.g., “d0026” is 26 dpi) and number of sequences are indicated on the right. All PNGSs that reached at least 10% frequency at any one time point are included. PNGS changes are color coded: blue indicates a gain, red a loss, and yellow transient changes. Gray indicates PNGSs that did not change substantially. (B–E) Glycan shields of CH152 Envs at transmission (TF) and subsequent time points. For the latter, PNGSs present in >50% of Env sequences from the time point were used to generate a consensus glycan shield for the respective time point. Colors as in Figure 1B. (F)ID50 neutralization titers of wild-type and mutant (N339 containing) CH152 TF Envs by 89- to 285-dpi plasmas. (G)ID50 titers of wild-type and mutant day 535 CH152 Envs by 1- to 2-year plasmas. (H) As in (G), but with a day 1,297 Env. See Table S4 for autologous ID50 titers.

To characterize the forces that drive glycan shield evolution, we examined the distribution of Env PNGSs in subject CH152, who developed the capacity to neutralize only 1/12 (8%) of the heterologous panel 4 years postinfection (Figure 3A). We calculated the frequency of each PNGS at each time point (Figure 4A) and approximated the consensus glycan shield by using PNGSs present in >50% Env sequences from that time point. These analyses revealed a substantial reduction of unshielded areas over time (Figures 4B–4E).

Subject CH152’s TF Env contained three glycan holes outside of hypervariable loops: at the base of the V3 loop, in C3, and in V4. The V3 hole was filled by serial addition of 3 PNGSs: N301, predominant by 285 days postinfection (dpi); N413 by 535 dpi; and N332 by 1,297 dpi (Figure 4A). The N339 glycan hole was mostly filled by 285 dpi. The N392 hole was sporadically and transiently filled and never reached fixation. Overall, the CH152 Env glycan hole area decreased from 4,385 Å^2^ at transmission to 494 Å^2^ in 3.5 years postinfection due to the above glycan acquisitions (Figure 4B–E).

An earlier study had provided direct evidence for the hypothesis that filling rare glycan holes could enable escape from glycan hole-targeting NAbs in natural infections: a hole near position 295 in subject CH40’s TF (Figure 3A) was filled over time, a change that reduced the TF sensitivity by 4.3- to 5.2-fold to 4- and 6-month plasma (Bar et al., 2012). To examine whether similar escape mechanisms drove glycan shield evolution in CH152, we tested the neutralization sensitivity of a panel of pseudoviruses containing native and glycan-mutated CH152 Envs to autologous plasma. The acquisition of the N339 glycan rendered the CH152 TF Env 2.2- to 3.1-fold more resistant to neutralization by plasmas collected within the first 10 months (Figure 4F; Table S4). Testing of Env variants from later time points (535 and 1,297 dpi) showed that the acquisition of N301 and N413 also rendered CH152 quasispecies members more resistant to plasma neutralization (Figures 4G and4H). The most striking effect was the removal of N413 from a day 1,297 Env, resulting in a 41-fold increase in neutralization sensitivity to plasma from day 698 (Figure 4H). Similarly, removal of N413 from a day 535 variant made it 2.6- to 16-fold more sensitive to day 439–698 plasmas; the subsequent removal of N301 increased sensitivity further by 1.6- to 2-fold (Figure 4G; Table S4).

PNGS additions did not always result in resistance and in some cases even increased Env sensitivity to plasma neutralization (Figures 4G and4H; Table S4). For example, addition of N301 rendered the CH152 TF Env 2-fold more sensitive to 89- to 145-day plasmas (Table S4). However, addition of N339 or N413 to TF+N301 restored the TF phenotype. Thus, the effect of glycan additions and losses can be complex and context dependent. Nonetheless, epitope mapping indicated that the two major predicted CH152 TF glycan holes (N339 and V3) were targeted by autologous NAbs.

### TF Glycan Holes Are under Positive Selection to Be Filled

**Figure 5.**
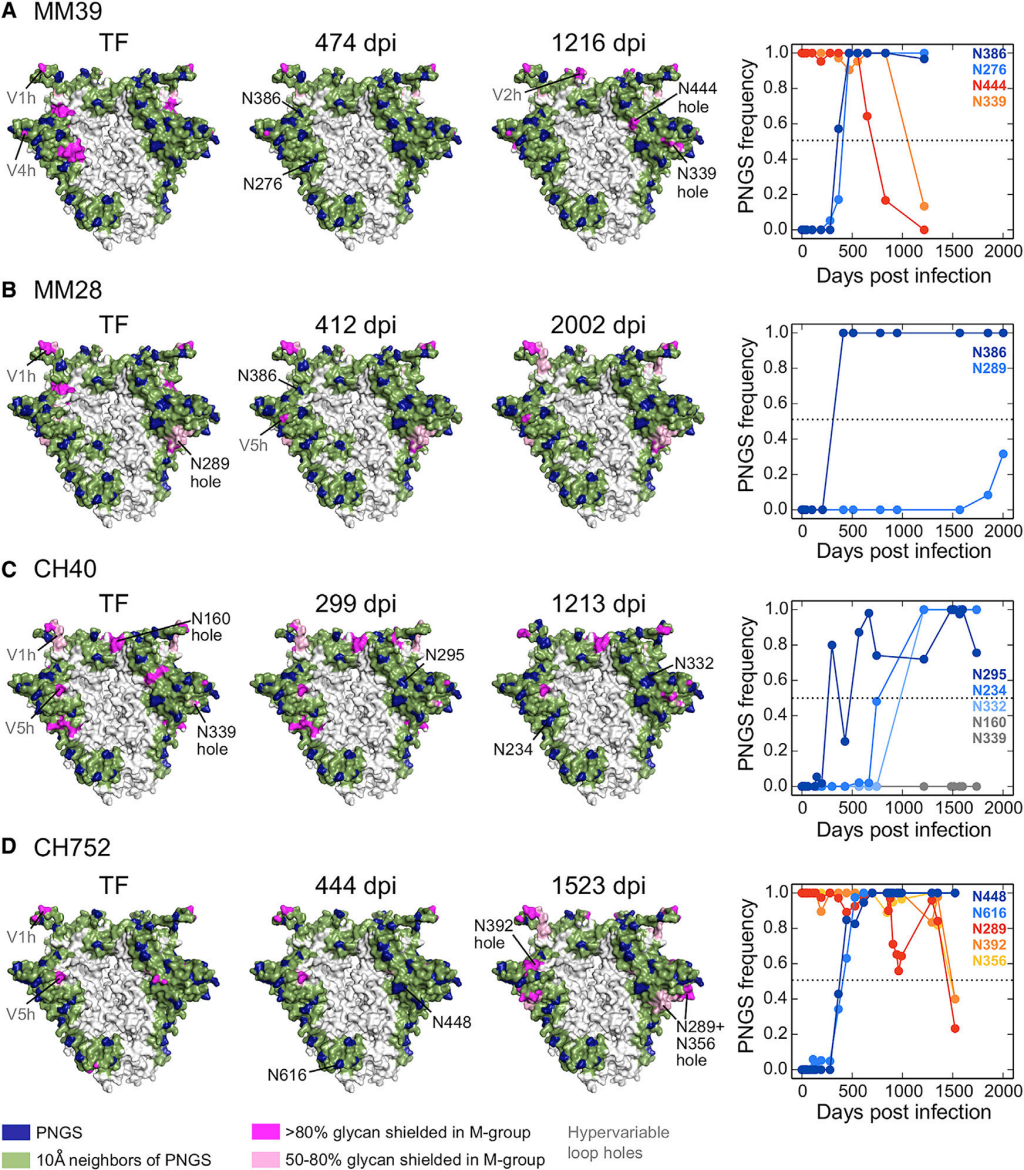
Glycan Shield Evolution in Individuals Who Developed Modest Heterologous Neutralization Breadth (A–D) Glycan shield evolution for individuals MM39 (A), MM28 (B), CH40 (C), and CH752 (D). Glycan shields are shown for Envs at transmission (TF) and for subsequent time points using consensus PNGSs at the time point; colors as in Figure 1B. Plots on the right depict the fraction of sequences with a particular PNGSs at each time point in each subject; all non-hypervariable loop PNGSs with frequency changes are shown. Dark and light blue curves show PNGS acquisitions, yellow and red curves PNGS losses, and gray curves unfilled glycan holes over time.

**Figure 6.**
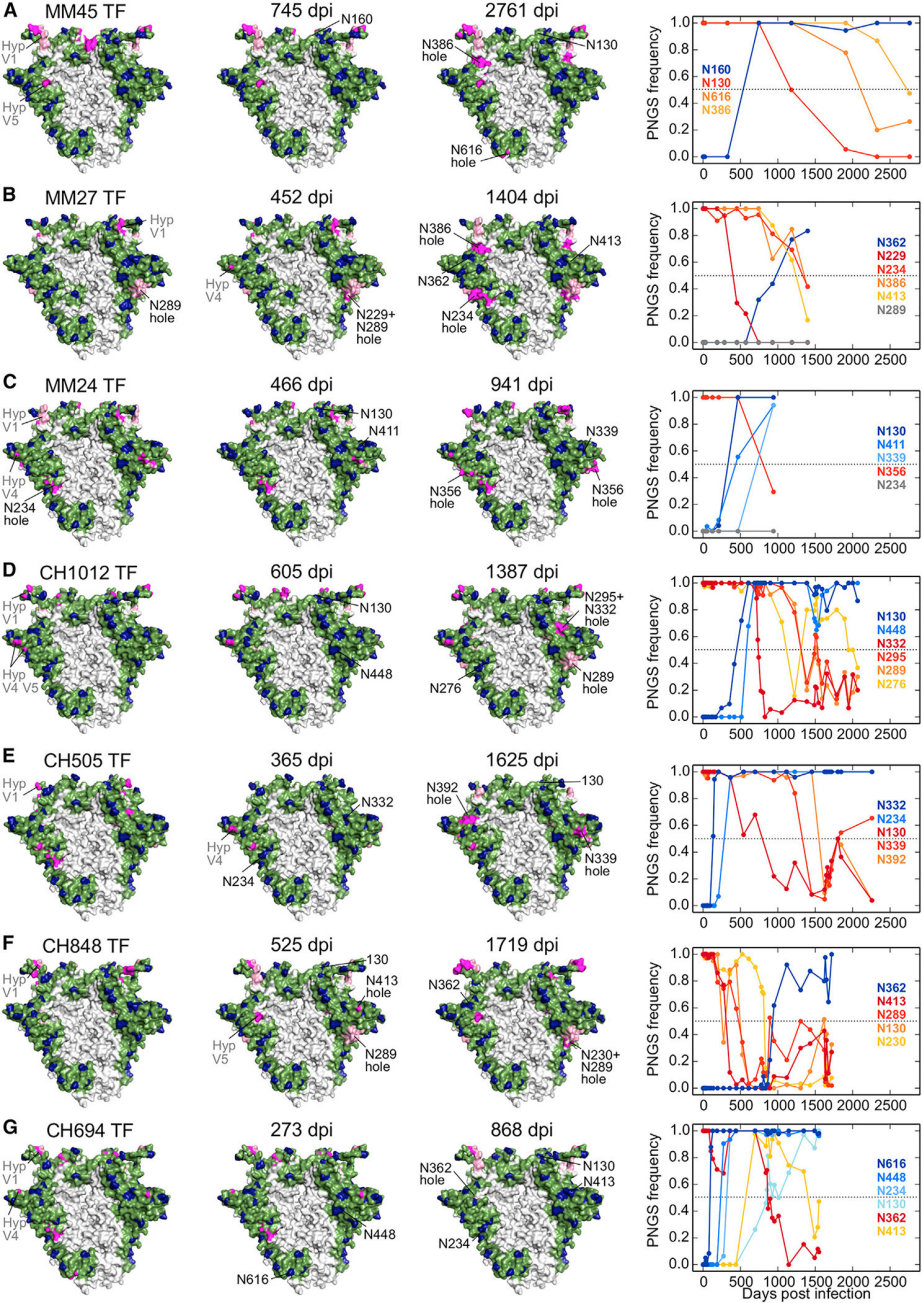
Glycan Shield Evolution in Individuals Who Developed Substantial Heterologous Neutralization Breadth (A–G) Same as Figure 5 except for individuals MM45 (A), MM27 (B), MM24 (C), CH1012 (D), CH505 (E), CH848 (F), and CH694 (G).

Analyzing glycan shield evolution in the remaining subjects showed that 16 of the 22 sizeable, non-hypervariable region glycan holes present at transmission in the 12 subjects were filled by subsequent PNGS acquisition mutations (Figures 5 and 6; summarized in Figure 3B). CH848 and CH1012 TFs had no sizeable glycan holes (Figure 3B), but in each a small TF glycan hole was filled (N362 in CH848, N130 in CH1012, Figure 6). Of the 10 subjects with larger TF glycan holes, all predicted holes were filled in 5 subjects (CH152, CH694, CH752, MM39, MM45), and 1–2 holes were filled in 4 others (CH40, CH505, MM24, and MM28) (Figures 4, 5, and 6). The single glycan hole found in MM27 TF, around position 289, was not filled at our cutoff of >50% at any time point; however, a PNGS at N289 was acquired at a low frequency at later time points (Figure 6B). Finally, while most PNGS acquisitions reduced the glycan hole area on the trimer surface, PNGS acquisitions at N130 in MM24, N332 in CH40, N362 in MM27, and N448 in both CH694 and CH1012 (Figures 5 and 6) failed to do so.

PNGS acquisitions that filled TF glycan holes with >50% frequency arose at different times: 8/16 (50%) in the first year of infection, 5 (31%) in the second year, and 3 (19%) in the third year. All but three of these acquired PNGSs were selected to fixation or maintained at high frequencies (Figures 4, 5, and 6), indicative of positive selection (Hraber et al., 2015).

To further explore whether the glycan holes were likely immune targets, we looked for evidence of positive selection at the amino acid level in the exposed regions. We identified all surface-exposed residues for each of 22 sizeable TF glycan holes and compared the rates of non-synonymous mutations to those of synonymous mutations at these sites during the first 3 years of infection. Non-synonymous mutations were significantly enriched in 10 of the 22 glycan holes, each of which were eventually filled (Table S5A; summarized in Figure 3B; see Figure S6 for non-synonymous mutation sites). Non-synonymous substitutions were also enriched in several of these glycan holes relative to the rest of the Env surface using a binomial test (Table S5B). Notably, for the low-breadth individuals CH152 and CH752, all TF glycan holes showed signs of positive selection.

We next re-examined the choice of the glycan-shielding radius for individual PNGSs (Figure S5). While 12- and 15-Å cutoffs yielded stronger correlations with neutralization breadth (Figure S5C), both failed to identify some important TF glycan holes that were captured using the 10-Å cutoff (Figures S5D and S5E). The 15-Å cutoff missed 10 of 16 glycan holes that were filled by PNGS additions, including the experimentally verified targets of autologous NAb responses, N339 hole in CH152 and N295 hole in CH40 (Figure S5D). The 12-Å cutoff performed better (Figure S5E), but still missed the glycan holes at N339 in the CH152 and MM24 TFs, and at N448 in the CH752 TF, which were filled over time and showed significant enrichment of local non-synonymous mutations (Figures 4, 5, and 6; Table S5). Thus, the 10-Å cutoff provided the most accurate glycan hole predictions based on regions known to be targeted by autologous NAbs as well as localized positive selection in longitudinal sequence data.

### Heterologous Neutralization Breadth Develops after Filling of Glycan Holes

**Figure 7.**
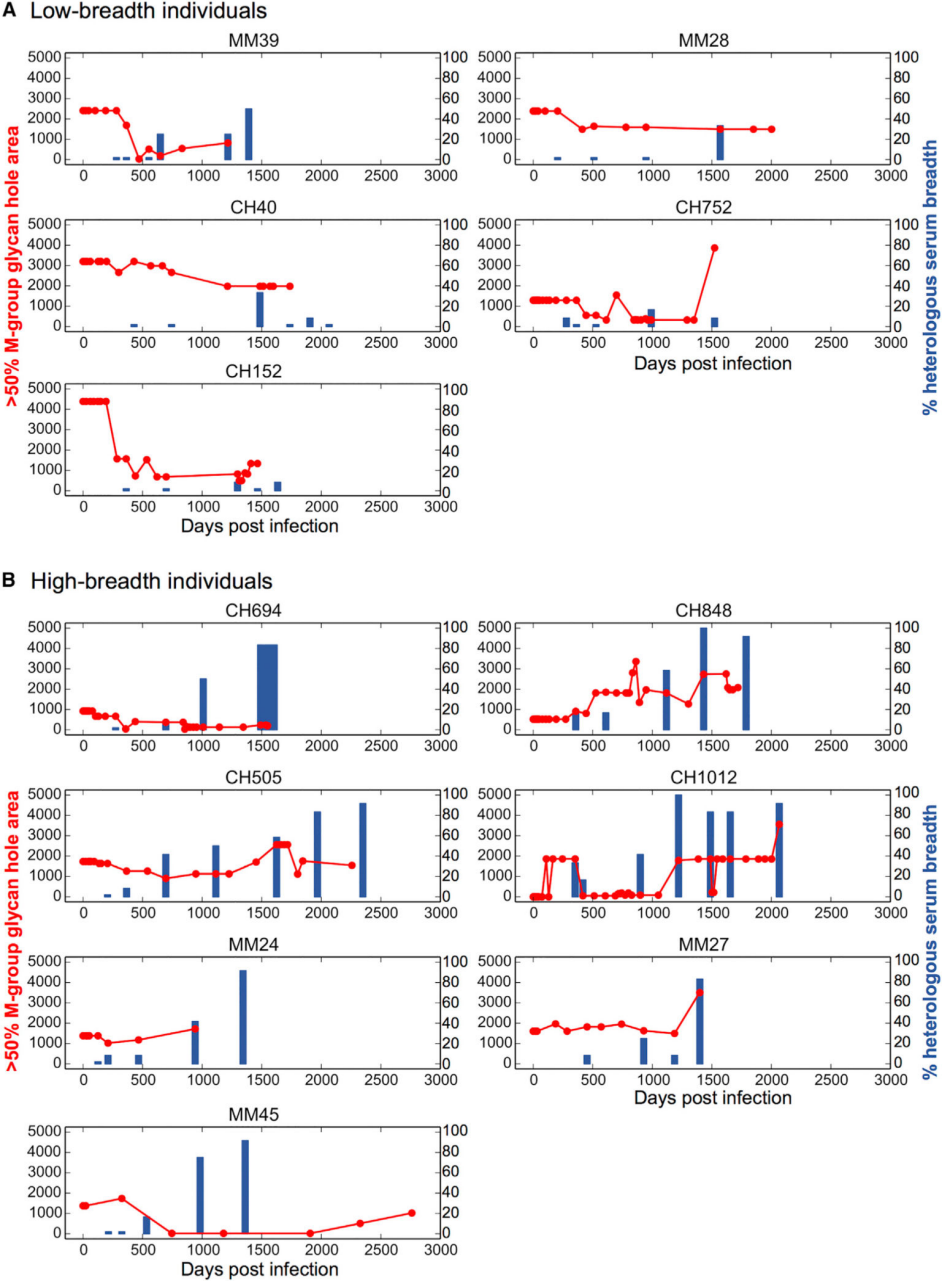
Kinetics of the Env Glycan Hole Area and Development of Heterologous Breadth (A) Data for low-breadth individuals. (B) Data for high-breadth individuals.For each subject, the consensus glycan hole area (red; using the >50% M-group conserved glycan shield) is shown in relation to neutralization breadth (blue bars; percentage of neutralization of 12-member global panel) over time (days postinfection).

We next compared the timing of the acquisition of plasma neutralization breadth relative to the evolution of glycan-shielded areas (Figure 7). In 4 of 5 individuals who developed limited neutralization breadth (MM39, MM28, CH40, CH152), TF glycan holes were either partially or completely filled before any heterologous tier 2 responses could be detected (Figure 7A). This was most clearly seen for subject MM39, where the glycan shield was almost completely restored prior to the emergence of modest heterologous breadth. For the other three individuals, partial or complete filling of glycan holes also preceded the onset of breadth, although a substantial lag was observed. Even for high-breadth individuals, such as CH694, CH505, MM24, and MM45, onset of neutralization breadth coincided with the filling of TF glycan holes (Figure 7B); however, the exact timing could not be determined. For the other high-breadth subjects, the TF Env glycan shield was either near complete (CH848 and CH1012) or contained glycan holes that exhibited no signs of early immune targeting (MM27) (Figure 6; Table S5).

Similar patterns were found in the infant BG505 (Goo et al., 2014). The BG505 TF Env had a large glycan hole near N241 and N289 (Figure S7) that when used as an immunogen elicited strong neutralizing responses that lacked breadth (Klasse et al., 2016; McCoy et al., 2016). In contrast, the infant developed potent broadly cross-reactive bNAbs (Goo et al., 2014). Evaluation of Env sequences from three time points (weeks 6 and 14, and month 27) revealed several PNGS acquisitions between week 14 and month 27 (Figure S7A), including N241 that partially filled the large TF glycan hole, and N332 that filled a small hole (Figures S7B and S7C). Since neutralization breadth was first observed at month 27, it is possible that bNAbs developed after the N241/N289 and/or the N332 glycan holes were partially filled.

These recurrent patterns across hosts indicate that neutralization breadth generally appears after or concurrent with viral evolution to fill TF glycan holes. This is consistent with the hypothesis that a more complete glycan shield may facilitate selection of antibodies with the potential to develop breadth. The pattern, however, may not be causative, as the sparse sampling times limit our ability to directly relate the timing of glycan-hole filling to neutralization breadth, and other factors correlated with acquisition of neutralization breadth (e.g., viral diversification and acquisition of antibody somatic mutations) also happen concurrently.

### Appearance of New Glycan Holes Later in Infection and Their Impact on Neutralization Breadth

In 8 of 12 subjects, new glycan holes arose at later time points (Figures 5, 6, and 7). The three subjects with no substantial TF glycan holes (CH1012 and CH848) or TF glycan holes that were never filled (MM27) acquired 1–2 new glycan holes beginning at 452–1,387 dpi (Figure 6). In the other five (MM39, CH752, MM45, MM24, CH505), 1–2 new glycan holes emerged, but only after the initial TF Env glycan holes were filled (Figures 5 and 6). The new glycan holes did not overlap with those present at transmission. For example, after TF glycan holes at N234 and N332 were filled, CH505 viruses exhibited PNGS deletions at positions 339 and 392 that resulted in new glycan holes (Figure 6E). While these PNGSs were lost from the majority of sequences from a given time point, most were never completely lost (Figures 5 and 6). Interestingly, the creation of new glycan holes was not associated with a reduction in breadth, but instead often coincided with increasing NAb breadth (Figure 7). It is possible that the lost glycans were targeted directly by Abs, or that they enabled epitope exposure indirectly and their removal facilitated immune escape. Thus, *in vivo* immune (or other) pressures not only fill but also create new unshielded Env areas, explaining the presence of glycan holes in many TF viruses.

## DISCUSSION

Uncommon glycan unshielded areas in HIV-1 Env vaccines are often targeted by NAbs that typically lack breadth (Bradley et al., 2016; Crooks et al., 2015; Klasse et al., 2016; McCoy et al., 2016; Pauthner et al., 2017; Sanders et al., 2015; Torrents de la Peña et al., 2017). Here, we show that glycan holes in TF viruses can also serve as targets for NAb responses in natural infection and are associated with delayed development of neutralizing breadth.

We developed a sequence- and structure-based computational approach to predict the three-dimensional glycan shield for a given Env (Figure 1). We used a 10-Å radius of protection around each PNGS, as it provided the best characterization of glycan holes targeted by immune responses (Figure S5). Factoring in Env trimer structure enabled us to account for the shielding by neighboring glycans. Because the method is computationally fast, the glycan shields of >4,500 globally sampled Env sequences could be calculated, providing a quantification of the Env glycan shield conservation across the majority of M group viruses. By identifying commonly shielded regions on the protein surface, we could visualize and quantify uncommon glycan holes specific for individual Envs.

Applying this approach, we discovered an Env-based correlate of neutralization breadth in natural infection - the size of the Env glycan holes in the transmitted virus was inversely correlated with the development of neutralization breadth (Figure 3). Analyses of the kinetics and quality of the neutralizing response in the context of glycan shield evolution yielded insight into potential mechanisms. First, the majority of TF Env glycan holes were likely targeted by autologous neutralizing responses (Figures 4, 5, and 6), which was experimentally confirmed in two subjects (CH40 in Bar et al., 2012 and CH152 in this study) and inferred for other subjects based on high rates of positive selection within the glycan holes. Second, viral escape from such responses in most cases resulted in the filling of glycan holes early in infection (Figures 4, 5, 6, and S6; Table S5). Third, the limited heterologous breadth that developed in low-breadth individuals tended to arise after most TF Env glycan holes were filled (Figure 7A), consistent with the idea that filling glycan holes may facilitate development of neutralization breadth. However, the lapse in time between filling glycan holes and onset of breadth varied widely between subjects, and the observed patterns may have been a surrogate for another time-dependent factor. The delay in the development of neutralization breadth associated with glycan holes could be due to immunodominant strain-specific responses that impede broader antibody lineages, similar to germinal center dominance of non-neutralizing responses in immunization studies (Havenar-Daughton et al., 2017). Additionally, the higher oligomannose content in the context of more heavily glycosylated proteins, resulting from glycan crowding inhibiting processing, could lead to improved NAb responses, as these glycoforms are associated with more effective presentation by dendritic cells (Behrens and Crispin, 2017; Jan and Arora, 2017; van Montfort et al., 2011), and some NAbs require oligomannose glycans for binding (MacLeod et al., 2016).

Although our mapping strategy revealed important relationships between glycan shield evolution and NAb responses, it has limitations. First, it does not account for variable glycan occupancy, although >90% PNGSs in SOSIP trimers are glycan occupied (Cao et al., 2017). Second, it does not account for glycoform heterogeneity, which varies both across sites and Envs (Behrens et al., 2016; Go et al., 2009, 2017). Thus, using a fixed 10-Å radius provides only an approximation of the contribution of a given glycan to the overall glycan shield. Third, glycans can extend ~20 Å perpendicularly from the Env protein surface (Gristick et al., 2016; Lee et al., 2016; Stewart-Jones et al., 2016). This spatial arrangement, which likely blocks antibody access to recessed inter-protomer regions, is not captured (Figures 1 and S1). Finally, due to the extreme sequence and length variation and a lack of structural resolution, glycan shielding within the hypervariable regions of the loops cannot be accurately mapped. Hypervariable regions tend to be glycan rich, but the placement and number of PNGS differs widely, both in the global population and within a host over time (Bonsignori et al., 2017; Gao et al., 2014; Korber et al., 2017). While these differences can impact bNAb sensitivity, they complicate systematic characterization of uncommon glycan holes in these regions.

BNAb development is a complex multi-factorial process and the completeness of the TF Env glycan shield represents only one of many factors known to impact bNAb development (Haynes and Verkoczy, 2014). For example, subject CH752 had a small TF Env unshielded area and a substantial viral load yet failed to develop neutralization breadth (Figure 3). It is possible that despite their small size, the holes in the CH752 TF were targeted by NAb responses, as they are filled and are under positive selection pressure; such responses could have delayed breadth development. However, other factors such as the pace of antibody/viral diversification, the level of T cell help, and host genetics may have impeded development of breadth. Moreover, targeting of glycan holes can be context and host dependent. For example, the N289 glycan hole in the MM27 TF virus exhibited no evidence of immune selection, while the same glycan hole in B41 SOSIP was consistently targeted in immunized rabbits (Klasse et al., 2016).

Our finding that a complete glycan shield at transmission favors the development of neutralization breadth during HIV-1 infection seems at odds with previous findings that removal of certain glycans facilitates Env binding to germline precursors of several bNAb classes, e.g., N276 for VRC01-class precursors (Stamatatos et al., 2017), N130 and N185 for V2 glycan bNAb precursors (Voss et al., 2017), and V1 glycans for PGT121 precursor (Steichen et al., 2016). Several factors can reconcile these findings with our results. First, not all bNAb precursors require glycan holes in autologous viruses for triggering, e.g., CH103 and CH235 precursors bound to CH505 TF that had a relatively complete glycan shield (Bonsignori et al., 2016), and CAP256 precursor neutralized the super-infecting autologous virus that had both N130 and a hypervariable V2 glycan (Bhiman et al., 2015). Second, while glycan-deficient Envs can trigger unmutated bNAb precursors, immunization with these alone may not be sufficient to guide subsequent bNAb lineage development. Instead, more completely glycan shielded Envs may be useful as follow-up immunogens to drive affinity maturation for broadening the initial germline response (Tian et al., 2016b). Third, glycans can provide stable anchoring as broad antibody lineages mature in the face of an evolving protein surface, as shown for the V2 apex CAP256 bNAb lineage (Andrabi et al., 2017). Fourth, intact shields with high glycan density may be enriched for high mannose sugars due to impaired processing by glycosidases (Behrens and Crispin, 2017), resulting in improved immune recognition for some antibodies. Fifth, due to glycan dynamics, access to the protein surface is hindered, but not completely blocked, even in Envs with a complete glycan shield. This likely explains the unusual features of HIV-1 bNAbs, such as long CDR3 regions and a tendency to require carbohydrate contacts (Burton and Hangartner, 2016; Kwong and Mascola, 2012; West et al., 2014).

In conclusion, we found that neutralization breadth development was limited in subjects with sizeable Env glycan holes at transmission. Our findings identify the Env glycan shield as a key determinant of bNAb development and suggest that Env immunogens with intact glycan shields may offer a more rapid route to neutralization breadth. The latter hypothesis is testable both in vaccination trials and SHIV-infected macaques.

## STAR⋆METHODS

### KEY RESOURCES TABLE

**Table:** 

REAGENT or RESOURCE	SOURCE	IDENTIFIER
Bacterial and Virus Strains		
HIV-1 SG3 Δenv	George Shaw (University of Pennsylvania); Wei et al., 2003	N/A
Stbl2 Competent Cells	Invitrogen	Cat#102680019
HIV-1 SG3Δenv/K101P.Q148H.Y181C	Michael Seaman (BIDMC)	N/A
Panel of Global HIV-1 Env Clones (n = 12)	NIH AIDS Reagents Program	Cat#12670

Chemicals, Peptides, and Recombinant Proteins		

DEAE Dextran	Sigma-Aldrich	Cat# D-9885
Human Buffy Coat, Fresh	ZenBio, Inc.	Item# SER-BC-SDS
DPBS, no calcium, no magnesium	GIBCO by Life Technologies	Cat# 14190250
Ficoll Paque Plus	GE Healthcare Life Sciences	Cat# 17144003
autoMACS Running Buffer	Miltenyi Biotec Inc.	Cat# 130091221
RPMI 1640 Medium	GIBCO by Life Technologies	Cat# 11875119
Fetal Bovine Serum-characterized	GE Healthcare Life Sciences	Cat# SH30071.03
Penicillin-Streptomycin-Glutamine (100x)	GIBCO by Life Technologies	Cat # 10378016
Fugene-6	Promega	Cat #E2692
Trypsin-EDTA	GIBCO by Life Technologies	Cat #25300120
CD4 MicroBeads, human	Miltenyi Biotec Inc.	Cat# 130045101
CryoStor® cell cryopreservation media CS5	Sigma-Aldrich	Cat# C2999
Proleukin® (aldesleukin)	Prometheus Therapeutics and Diagnostics	Item# 147874 at the Hospital at the University of Pennsylvania Pharmacy
HIV Infectivity Enhancement Reagent	Miltenyi Biotec Inc.	Cat# 130095093
SuperScript III Reverse Transcriptase	Invitrogen	Cat# 18080044
10mM dNTP mix	Invitrogen	Cat# 18427088
RNaseOUT Recombinant Ribonuclease Inhibitor	Invitrogen	Cat# 10777019
Platinum Taq DNA Polymerase High Fidelity	Invitrogen	Cat# 11304011
Ribonuclease H	Invitrogen	Cat# 18021014
Ethyl alcohol, Pure (200 proof)	Sigma-Aldrich	Cat#E7023–500ML
AMPure XP Beads	Beckman Coulter	Cat#A63880
LB Broth (Miller)	Sigma-Aldrich	Cat#L3522
LB Agar	Invitrogen	Cat#22700–025
Ampicillin	ThermoFisher	Cat#11593027

Critical Commercial Assays		

Luciferase Assay System	Promega	Cat# E1500
HIV p24 (high sensitivity) AlphaLISA Detection Kit	Perkin Elmer	Cat# AL291F
Nextera DNA Sample Preparation Kit (96 samples)	Illumina	Cat#FC-121–1031
Nextera Index Kit (96 indices, 384 samples)	Illumina	Cat#FC-121–1012
Library Amplification Kit – Standard Kit (250 X 50ul reactions)	KAPA Biosystems	Cat#KK2612
MiniSeq Mid Output Kit (300 cycles)	Illumina	Cat#FC-420–1004
MiSeq Reagent Micro Kit v2 (300 Cycles)	Illumina	Cat#MS-103–1002
T Cell Activation/Expansion Kit, human	Miltenyi Biotec Inc.	Cat# 130091441
EZ1 virus mini kit v2.0	QIAGEN	Cat# 955134
BigDye Terminator v3.1 Cycle Sequencing Kit	ThermoFisher Scientific	Cat#4337455
Qubit dsDNA HS Assay Kit (500 assays)	Invitrogen	Cat#Q32854
QIAquick PCR Purification Kit	QIAGEN	Cat#28104
QuikChange II XL Site-Directed Mutagenesis Kit	Agilent Technologies	Cat# 200521

Experimental Models: Cell Lines		

Human HEK293T cells	ATCC	Cat# CRL-11268; RRID: CVCL_0063
Human TZM-bl cells	ATCC	Cat# PTA-5659
Oligonucleotides		

Primer: RT Primer Reverse: ACTACTTGAAGCACTCAAGGCAAGCTTTATTG	Salazar-Gonzalez et al., 2009	N/A
Primer: miSeq Sequencing primer P1: AATGATACGGCGACCACCGA	Iyer et al., 2017	N/A
Primer: miSeq Sequencing primer P2: CAAGCAGAAGACGGCATACGA	Iyer et al., 2017	N/A
Primer: N339 mutagenesis primer Forward: GAACTGACTGGAATGAAA CTTTACAAGGGGTAGG	This paper	N/A
Primer: N339 mutagenesis primer Reverse: CCTACCCCTTGTAAAGTTT CATTCCAGTCAGTTC	This paper	N/A
Primers for single genome amplification and Sanger Sequencing, see Table S6	This paper; Jones et al., 2004; Parrish et al., 2012; Salazar-Gonzalez et al., 2008, 2009; Sauter et al., 2009	N/A

Recombinant DNA		

pcDNA3.1(+)-CH152.env.TF	GenScript/This paper	N/A
pcDNA3.1(+)-CH152.env.TF.a301	GenScript/This paper	N/A
pcDNA3.1(+)-CH152.env.TF.a301a413	GenScript/This paper	N/A
pcDNA3.1(+)-CH152.env.TF.a301a413s332	GenScript/This paper	N/A
pcDNA3.1(+)-CH152.env.TF.a339	This paper	N/A
pcDNA3.1(+)-CH152.env.TF.a301a339	This paper	N/A
pcDNA3.1(+)-CH152.env.d535	GenScript/This paper	N/A
pcDNA3.1(+)-CH152.env.d535.s332	GenScript/This paper	N/A
pcDNA3.1(+)-CH152.env.d535.d413	GenScript/This paper	N/A
pcDNA3.1(+)-CH152.env.d535.d413d301	GenScript/This paper	N/A
pcDNA3.1(+)-CH152.env.d1297	GenScript/This paper	N/A
pcDNA3.1(+)-CH152.env.d1297.s334	GenScript/This paper	N/A
pcDNA3.1(+)-CH152.env.d1297.s334d413	GenScript/This paper	N/A
pcDNA3.1(+)-CH152.env.d1297.s334d413d301	GenScript/This paper	N/A
pHit456	Alan Kingsman (University of Oxford); Soneoka et al., 1995	N/A

Software and Algorithms		

Gen5 Data Analysis Software	BioTex	https://www.biotek.com/; Part#: GEN5
Geneious	Kearse et al., 2012	https://www.geneious.com; RRID: SCR_010509
RAxML version 8.2.4	Stamatakis, 2014	https://github.com/stamatak/standard-RAxML; RRID: SCR_006086
ClustalW version 2	Larkin et al., 2007	https://www.ebi.ac.uk/Tools/msa/clustalw2/; RRID: SCR_002909
FigTree version 1.4.3	A. Rambaut	http://tree.bio.ed.ac.uk; RRID: SCR_008515
PyMOL version 1.7.2 (Mac)	Schödinger LLC	https://www.pymol.org; RRID: SCR_000305
Poisson-Fitter	Giorgi et al., 2010	https://www.hiv.lanl.gov/content/sequence/POISSON_FITTER/pfitter.html
SNAP	B. Korber	https://www.hiv.lanl.gov/content/sequence/SNAP/SNAP.html
R version 3.3.2	R core team	http://www.R-project.org
Python	http://www.python.org	RRID: SCR_008394
SciPy	http://www.scipy.org	RRID: SCR_008058

Deposited Data		

HIV-1 sequences from this paper	This paper	GenBank: MF352844 to MF353070 and MG897823 to MG902890

Other		

E-Gel 96 Agarose Gels, 1%	Invitrogen	Cat# G700801
Performa DTR V3 96-Well Short Plates	EdgeBio	Cat#80808
High Sensitivity D1000 ScreenTape	Agilent	Cat#5067–5584
High Sensitivity D1000 Reagents	Agilent	Cat#5067–5585

### CONTACT FOR REAGENT AND RESOURCE SHARING

Further information and requests for reagents should be directed to and will be fulfilled by the Lead Contact, Bette Korber (btk@lanl.gov).

### EXPERIMENTAL MODEL AND SUBJECT DETAILS

#### Human subjects

Subjects were either enrolled in the Duke Center for HIV/AIDS Vaccine Immunology (CHAVI) 001 acute infection cohort (CH40, CH152, CH505, CH694, CH752, CH848, and CH1012; from United States (subtype B, n = 1), Malawi (subtype C, n = 5), or South Africa (subtype C, n = 1)) or recruited from the Mortimer Market Centre clinic in the United Kingdom (MM24, MM27, MM28, MM39, and MM45; all subtype B). Peripheral blood sampling was initiated following presentation with symptoms consistent with acute retroviral syndrome and regularly performed for 4–9 years of infection in the absence of antiretroviral treatment (Tables S1 and S2). Subjects provided informed consent and were offered antiretroviral therapy based on the standard of care at the time of trial enrollment. Subjects underwent regular clinical evaluations and viral load and CD4+ T cell count determinations (Table S2) until they were lost to follow up or initiated ART, with six individuals returning for 1–2 clinic visits post ART initiation. CH694 took a single dose of nevirapine when she delivered a baby on February 11, 2009 per standard of care practices (Table S2). Approval for CHAVI 001 and Mortimer Market Centre study protocols was obtained from the Duke Institutional Review Board and The National Health Service Camden/Islington Community local Research Ethics Committee, respectively.

### METHOD DETAILS

#### Glycan shield mapping strategy

##### Baseline Strategy

For a given sequence, the positions of PNGS were identified as an NXS or NXT amino acid sequence motif (where X is not Pro). We assumed that each PNGS is glycan occupied, except for overlapping PNGS (e.g., NNSS), where the last sequon was arbitrarily assumed to be glycosylated. These sites were then mapped on to the Env trimer structure for X1193 (subtype G, PDB: 5FYJ) (Stewart-Jones et al., 2016). If the hypervariable loops for a given Env were smaller than the corresponding loops in X1193, then the extra residues from the middle of the hypervariable loops from the X1193 structure were not shown. In case of longer hypervariable loops, all the hypervariable loop PNGS for the Env of interest were mapped on the crystal structure by using nearby sites available in the crystal structure. The glycan shield was then calculated by selecting all the trimer atoms in the structure that fell with 10Å of the alpha carbon atom of the PNGS asparagine using the “around” function in PyMOL (The PyMOL Molecular Graphics System, Version 1.7.2 Schrödinger LLC). To calculate glycan shields using other glycan shielding distance cutoffs, the same procedure as above was followed with the exception of using the cutoff of interest instead of 10Å.

##### M group conservation of glycan shielding

The 2015 HIV-1 Env Filtered Web alignment from the Los Alamos Database was used (n = 4,582 sequences including circulating recombinant forms, with one sequence per individual). Since the above detailed glycan shield mapping was not possible for such large number of sequences, we automated this calculation as follows. We first coded the handling of crystal structure coordinates and 3D distance calculation in Python. We next calculated the mapping between the M-group alignment position and the corresponding site on the crystal structure. Using this map, the PNGS for a given Env in the M-group alignment could be mapped on to the crystal structure, and using the above Python code, the glycan-shielded atoms for this Env could be found. Due to the extensive length variation and lack of sequence alignment in hypervariable regions, the PNGS in these regions could not be mapped using the above strategy. For this we used the observation that on an average 1 out 7 positions in hypervariable are PNGS (data not shown) and assumed that each hypervariable PNGS will shield up to 3 amino acids in the linear sequence on either side. This allowed assigning an approximate glycan shielding for the atoms in the hypervariable loops. Pooling together the data for each atom in the crystal structure that was or was not glycan shielded for each Env in the M-group alignment, we calculated the fraction of M-group Envs in which a given atom in the crystal structure is glycan shielded. The PDB file with fraction M-group glycan shield conservation loaded in the b-factor column is provided in Data S1.

##### Final Strategy

We next used PyMOL to calculate the glycan shield of a given Env of interest using the baseline strategy mentioned above, and overlaid this on the 50%–80% and > 80% M-group conserved glycan shields. The two cutoffs for consistently glycan-shielded atoms in M-group were based on Figure 1A right, and selected prior to our analysis of the virus from the 12 longitudinally studied subjects. Both cutoffs were applied to the sequences from the 12 subjects, and both yielded significant negative correlations between TF glycan hole area and breadth (Figures 3C and S4A). The infrequent glycan holes were characterized by calculating the solvent-exposed surface area (SASA) for the atoms that were not glycan shielded for a given Env, but were glycan shielded in most M-group Envs (at > 50% or other specified conservation cutoffs). SASA was calculated using the “get_area” function in PyMOL with the following parameters: dot_solvent = on, dot_density = 4, solvent_radius = 1.4. Due to the uncertainty in mapping the glycan shield in hypervariable regions, they were excluded for area calculations. For the visualization and quantification of glycan holes for longitudinal sequences, the above procedures were used with PNGS that were in 50% or more Env sequences from each time point for each subject. A webtool implementing this strategy is under development and will be made available at https://hiv.lanl.gov/content/sequence/GLYSHIELDMAP/glyshieldmap.html.

##### Glycan shield calculation for BG505-Δgly4 variants

To directly compare out results to those reported in Zhou et al., 2017, we used the following modified strategy to calculated glycan unshielded area for BG505-Δgly4 variants. We first used the crystal structure of BG505-Δgly4 (PDB: 5V7J, Zhou et al., 2017). We then used the baseline strategy above to estimate the glycan shielded area with the exception that hypervariable regions were not excluded, to match the analysis of Zhou et al. (2017). We note that only one other immunogen in Zhou et al. (2017), 426c-Δgly4, showed a significant correlation between autologous titers and glycan unshielded area for the glycan knock-in variants. We did not attempt the above calculation for this immunogen because there is no structural information available on this strain and thus, estimating the glycan shielded area due to the addition of a glycan in hypervariable region V5 based on other structures would be inaccurate.

##### Glycan shield calculation using BG505 crystal structure

Same strategies for baseline mapping of glycan shields, calculation of M-group glycan shield conservation and calculation of infrequent TF glycan hole areas were employed with the exception that the BG505 trimer crystal structure from PDB: 5FYL (Stewart-Jones et al., 2016) was used as the reference structure instead of the X1193 structure mentioned above.

#### Single Genome Sequencing

Single genome amplification from plasma viral RNA and direct amplicon sequencing was performed as previously described (Keele et al., 2008; Salazar-Gonzalez et al., 2009). Briefly, ~20,000 copies of viral RNA were extracted from plasma (QIAGEN, QIAamp Viral RNA kit) and reverse transcribed using SuperScript III Reverse Transcriptase (Invitrogen) in the presence of 0.5 mM dNTPs, 5 mM DTT, 2 units/μl RNaseOUT and 10 units/μl reverse transcriptase. Viral cDNA was then endpoint diluted and amplified using nested PCR. For CH40, CH152, CH505, CH694, CH752, CH848, CH694, and CH1012, PCR primers were as follows: first round forward primer 5′-CAAATTAYAAAAATTCAAAATTTTCGGGTTTATTACAG-3′; first round reverse primer 5′-ACTACTTGAAGCACTCAAGGCAAGCTTTATTG-3′; second round forward primer 5′-GGGTTTATTACAGRGACAGCAGAG-3′; and second round reverse primer 5′-GCACTCAAGGCAAGCTTTATTGAGGCTTA-3′. For MM24, MM27, MM28, MM39, and MM45 PCR primers were as follows: first round forward primer 5′-TAGAGCCCTGGAAGCATCCAGGAAG-3′; first round reverse primer 5′-TTGCTACTTGTGATTGCT CCATGT-3′; second round forward primer 5′-TAGGCATCTCCTATGGCAGGAAGAAG-3′; and second round reverse primer 5′-GTCTCGAGATACTGCTCCCACCC-3′. PCR reactions were performed in the presence of 2mM MgSO_4_, 0.2 mM dNTPs, 0.025 units Platinum Taq Hi-fidelity Polymerase/μl (Invitrogen), 0.2 μM forward primer, 0.2 μM reverse primer and endpoint-diluted cDNA. First round PCR conditions were as follows: denaturation at 94 C for 2 min; 35 cycles of denaturation at 94 C for 15 s, annealing at 55 C for 30 s, and extension at 68 C for 5.5 min (for CH40, CH152, CH505, CH694, CH752, CH848, CH694, and CH1012) or 3.5 min (for MM24, MM27, MM28, MM39, and MM45); extension at 68 C for ten min. The only modification to PCR conditions for the second reaction was a decrease in extension time to 5 min (for CH40, CH152, CH505, CH694, CH752, CH848, CH694, and CH1012) or 3 min (for MM24, MM27, MM28, MM39, and MM45). Second round PCR amplicons were then directly sequenced using either Sanger Sequencing or the Illumina MiSeq platform. Sequences with ambiguous bases were discarded.

#### Generation of limiting dilution HIV-1 isolates

Plasma samples were end-point diluted and used to infect 1×10^6^ activated CD4 T cells isolated from multiple donors divided into 24-well plates (Iyer et al., 2017). Cultures were maintained for 20 days, tested for p24 antigen (Parrish et al., 2013), and p24-positive wells were further expanded in fresh CD4+ T cells for an additional 10 days.

#### Isolate sequencing

Viral RNA was extracted from culture supernatants, reverse-transcribed, and the resulting cDNA used to amplify 3′ genome halves as described above (Parrish et al., 2013; Salazar-Gonzalez et al., 2009). miSeq libraries were prepared and sequenced as previously described (Iyer et al., 2017).

#### Heterologous Neutralization

Patient plasma samples were tested for neutralization activity using the luciferase-based reporter assay in TZM.bl cells as previously described (Montefiori, 2009; Sarzotti-Kelsoe et al., 2014). Samples were assayed using a primary 1:20 dilution and 3-fold titration series in duplicate wells, and the plasma dilution that inhibited 50% or 80% of virus infection (ID50 and ID80, respectively) was calculated. Some samples were 2-fold diluted, in which case titers were scaled to account for this (e.g., primary dilution 1:40). Neutralization activity was tested using the global reference panel of 12 HIV-1 Env pseudoviruses (deCamp et al., 2014), and murine leukemia virus (MuLV) served as negative control virus. Env pseudoviruses were produced as previously described (Li et al., 2005). For testing of plasma samples obtained when patients were on anti-retroviral therapy (ART), Env pseudoviruses were produced using an ART-resistant backbone vector that reduces background inhibitory activity of antiretroviral drugs present in the plasma sample (SG3ΔEnv/K101P.Q148H.Y181C, M.S.S., unpublished data). These assays were performed in a laboratory meeting GCLP standards.

#### Autologous Neutralization

PNGS additions that altered CH152 TF glycan holes were observed at days 285, 535, and 1297 post-infection. To test the impact of these glycan changes on autologous plasma neutralization, three naturally-occurring Env variants were synthesized (GenScript, Piscataway, NJ) and cloned in a pcDNA3.1 vector (Kraus et al., 2010). The first was the inferred CH152 TF Env, which elicited the earliest NAb response. The second was a naturally occurring day-535 variant, termed d0535.ipe026.2.16, that included a PNGS at position 413 and fell within a phylogenetic “rake” of genetically near-identical sequences. Phylogenetic rakes have previously been used to identify replication-competent viruses from plasma samples collected during chronic infection when sequences often contain inactivating mutations (Parrish et al., 2012). The third variant, termed d1297.ipe026.15.08, was selected from day 1297 sequences as the variant that encoded the PNGS shift from 334 to 332 and exhibited the shortest branch length to the TF in a maximum-likelihood phylogenetic tree (Figure S3) (Keele et al., 2008). Site-directed mutagenesis was then used to add or remove PNGS at positions 301, 339, 332, 334, and/or 413.

Individual *env* clones were co-transfected with HIV-1 SG3 Δenv backbone in 293T cells to generate Env-pseudotyped viruses and tested for autologous plasma neutralization in the TZM-bl neutralization assay (Barbian et al., 2015; Montefiori, 2009). Briefly, 5-fold serial dilutions of heat-inactivated CH152 plasma (1:20 – 1:12500) were incubated with 4,800 infectious units of virus in the presence of DEAE-dextran (40 μg/ml) before adding to TZM-bl cells in duplicate. 48 hours later, TZM-bl cells were analyzed for luciferase expression using a microplate reader. Relative infectivity was calculated by dividing the number of luciferase units for plasma dilutions with values obtained for wells that contained normal human plasma. Half-maximal inhibitory dilutions (ID_50_) were calculated using the mean relative infectivities of two independent assays and linear regression analysis. MuLV was used as a negative control.

### QUANTIFICATION AND STATISTICAL ANALYSIS

#### Estimating TF sequence and the time of infection

Using statistical modeling, phylogenetic trees and highlighter plots, as previously described (Keele et al., 2008), we characterized patterns of sequence diversity in early time point samples and established that all 12 subjects in this study had been infected by a single TF. Under this scenario, the consensus sequence from the first time point alignment is considered to be the most recent common ancestor of the infecting lineage, and mutations away from such sequence can be used to time the infection using a Poisson model of random accumulation of mutations (Giorgi et al., 2010; Keele et al., 2008). Based on these assumptions, we estimated the TF of each infection to be the consensus sequence,which we calculated using the LANL tool Consensus Maker (https://www.hiv.lanl.gov/content/sequence/CONSENSUS/consensus.html), and estimated the time since infection using the tool Poisson Fitter (https://www.hiv.lanl.gov/content/sequence/POISSON_FITTER/pfitter.html). The assumptions of the Poisson model were consistent with the data from all subjects (Goodness of Fit p value > 0.05), with the exception of subject MM27 (Table S1). The TF sequence and time since infection estimates were used in Figures 3, 4, 5, 6, 7, and S2–S6 and Tables S1, S2, S3, and S4

#### Phylogenetic analyses

Sequences alignments were produced using CLUSTALW version 2 (Larkin et al., 2007) followed by manual curation; hypermutated regions and sites that could not be unambiguously aligned were removed from subsequent phylogenetic analyses. Phylograms were constructed with RAxML version 8.2.4 using a GTRGAMMA model (Stamatakis, 2014). These data are shown in Figures S2 and S3.

#### Quantification of infrequent glycan holes

Infrequent glycan holes were quantified using SASA of the non-glycan-shielded atoms as described above in Method details. These analyses were used in Figures 3, 7, S4, and S5.

#### Sequence-only based approach for identifying infrequent glycan holes

As a comparison to the above sequence- and structure-based strategy, we also used the following strategy for identifying infrequent glycan holes using sequence alone. We first defined conserved PNGS based on the 2015 HIV-1 Env Filtered Web alignment from the Los Alamos Database. First, we ranked each residue position based on the PNGS prevalence in a 7-mer window, and then removed all positions with frequency < 50%. Then we took the highest rank position as a conserved glycosylation position, removed the 7-mer centered at this position from the list, and did this recursively to obtain a final list of conserved glycan positions. These were HXB2 positions 88, 130, 135, 142, 147, 156, 160, 187, 197, 234, 241, 262, 276, 289, 295, 301, 332, 339, 356, 386, 392, 398, 402, 411, 448, 463, 467, 611, 616, 625 and 637. The number of glycans holes for each test sequence using this strategy was defined as the number of conserved glycosylation positions where there is no PNGS within the 7-mer window. These analyses were used in Figure S4C.

#### Plasma Neutralization breadth

For heterologous plasma neutralization, some samples showed detectable reactivity to the negative control MuLV as measured by ID50 titers above the experimental thresholds of 20 or 40 (Table S3). To identify detectable neutralization of heterologous HIV strains by subject plasmas while accounting for such background, we used the criterion that ID50 against HIV strains should be higher than twice the MuLV ID50 titer. MuLV titers below experimental thresholds of 20 or 40 were set to 20 or 40, respectively. This quantification of breadth is used in Figures 3, 7, S4, and S5.

Estimates of neutralization breadth are sensitive to thresholds set to define positive neutralization. To confirm that our primary result showing the negative correlation between neutralization breadth and the glycan hole area of TF viruses (Figure 3B) was robust, we explored the impact of using two additional criteria: a more stringent cutoff that required HIV ID50 greater than 3-fold MuLV ID50, and a more relaxed one that required HIV ID50 greater MuLV ID50 + 10 (Table S3). Use of either of these criteria yielded significant negative correlations between infrequent TF glycan hole area (using > 50% M-group conserved glycan shield) and maximum heterologous breadth: HIV titer > MuLV titer + 10 gave p = 0.0015 (Kendall Tau rank test) and HIV titer > 3X MuLV titer gave p = 0.0085 (Figures S4B and S4C).

#### Set-point viral loads

Set-point viral loads were calculated using longitudinal plasma RNA copy data for 14–36 samples from each subject (Table S2) and using the method from (Fellay et al., 2007). Briefly, this method entails retaining those viral load data points that fall after the initial peak viremia and before the late increase in viral load that indicates onset of symptoms and progress to AIDS. Due to measurement errors etc., significant outliers among the retained viral load data points are removed (0.5 Log_10_ higher or lower viral load than the average of other retained data points). The set point viral load is then calculated by taking the geometric mean of the remaining viral load data points. These viral load data are used in Figure 3.

#### Correlation between infrequent TF glycan hole area and heterologous neutralization breadth

Kendall’s Tau rank test and Pearson R correlation test (as implemented in R version 3.3.2 (The R Foundation for Statistical Computing https://www.r-project.org/)) were used to quantify the statistical significance of the correlation between infrequent glycan hole area and maximum heterologous breadth (Figures 3, S4, and S5), and between set point viral loads and maximum heterologous breadth (Figure 3C). For bivariate modeling of maximum breadth using infrequent TF glycan hole area and set point viral loads, a generalized linear model was employed using the ‘glm’ function in R using the logistic link function and the binomial family for deviance. The model without interactions was better than the model with interactions using the Akaike Information Criterion (AIC) (difference in AIC = 1.85). The glm function estimates the statistical significance of the contribution of each variable using a t-test. This bivariate model was compared to the model of maximum breadth using TF glycan hole area alone, and the latter model was better using AIC (difference in AIC = 1.84)

#### Enrichment of non-synonymous mutations in TF glycan holes

For each subject, using the glycan shield mapping strategy above, the surface amino acids in each glycan hole in each TF were identified. All amino acids that had at least a single surface exposed atom (SASA > 0.0, calculated as above) in the TF glycan hole were considered, and those in hypervariable regions were excluded. For each glycan hole, the longitudinal Env sequences from the first 3 years were compared to the TF from the respective subject to identify synonymous and non-synonymous mutations at the glycan hole surface-exposed sites, using the program SNAP at the Los Alamos HIV Database (https://www.hiv.lanl.gov/content/sequence/SNAP/SNAP.html). The time frame of 3 years was chosen since most TF glycan holes were at least partially filled by this time (Figures 4, 5, and 6). The statistical significance of difference between non-synonymous and synonymous mutations was estimated with a Fisher’s exact test using the numbers of observed and total potential non-synonymous and synonymous mutations as computed in SNAP (if Nd (Sd) were the observed non-synonymous (synonymous) mutations and N (S) were total potential non-synonymous (synonymous) mutations, then the contingency table [[Nd, N-Nd],[Sd, S-Sd]] was analyzed using Fisher’s exact test). To eliminate the possibility that glycan acquisition mutations could be reversion to M-group consensus, arising for fitness reasons, we repeated the above calculations by excluding the sites that harbored PNGS acquisition mutations for each glycan hole. These mutations could be at the position of the PNGS that acquires a mutation to Asn, or 2 amino acids downstream that acquire mutation to Ser/Thr. The above analysis was repeated using the set of reduced sites for each TF glycan holes. We note that this latter approach is more conservative as it cannot detect selection at glycan holes where the acquisition of the PNGS was early and was sufficient in conferring escape from hole-targeting antibody responses. The results from these analyses are shown in Figure S6 and Table S5A.

We also did a comparison to simply evaluate the number of sites with non-synonymous mutations that occur in the holes versus outside the holes using a binomial test (Table S5B). For each subject, the fraction of Env sites that mutate away from the TF sequence in the first 3 years was calculated. Only those sites that were on Env surface and had at least 2 mutations away from TF in any time point were considered (to reduce the chance of potential sequencing artifacts), while sites within the hypervariable regions were not considered. By comparison of such mutating sites in the TF glycan holes to the overall fraction of mutated sites across Env the statistical significance of enrichment was found using a binomial test (as implemented in the ‘stats’ module in SciPy) one-sided p-value. These results are shown in Table S5B. This analysis supported the conclusions of the test for regional positive selection within the holes, but we feel it may be subject to some bias due to dramatic differences in selection pressures between different regions in Env, e.g., strong negative selection at interior sites and functional sites such as receptor binding site may bias the analysis.

### DATA AND SOFTWARE AVAILABILITY

Most of the sequences from the subjects CH505 and CH848 were reported earlier (Liao et al., 2013; Gao et al., 2014; Bonsignori et al., 2017), but a small number of CH505 sequences were added for this study (GenBank: MF352844 - MF353070). The sequences from the 10 new subjects from this study have been submitted (GenBank: MG897823 – MG902890). A full alignment of all sequences used in this study is available the “special interests alignments” section of the Los Alamos Database (https://www.hiv.lanl.gov/content/sequence/HIV/SI_alignments/datasets.html). Subject information and neutralization data are provided in Tables S1, S2, S3, and S4. The PDB file with M-group conservation of glycan shielding is provided in Data S1. We are also in the process of developing a web tool called “Glycan Shield Mapping” on the Los Alamos HIV Database (https://hiv.lanl.gov/content/sequence/GLYSHIELDMAP/glyshieldmap.html), which will provide glycan shield predictions for user provided Env sequences.

## Supplementary Material

1

9

10

2

3

4

5

6

7

8

## Highlights

- Computational strategy for prediction of infrequent HIV-1 Env glycan holes
- Intact glycan shields at transmission are associated with neutralization breadth
- Glycan holes fill *in vivo* to escape autologous neutralizing antibodies
- Filling of glycan holes precedes development of neutralization breadth
