# Supplementary material for: Completeness of HIV-1 Envelope Glycan Shield at Transmission Determines Neutralization Breadth
Source: Cell Rep. Author manuscript; Available in PMC 2019 Mar 20. (PMC6426304; doi:10.1016/j.celrep.2018.09.087)
Supplement: 1 [file NIHMS1511530-supplement-1.pdf]

Cell Reports, Volume *25*

Supplemental Information

Completeness of HIV-1 Envelope Glycan Shield at Transmission Determines Neutralization Breadth

Kshitij Wagh, Edward F. Kreider, Yingying Li, Hannah J. Barbian, Gerald H. Learn, Elena Giorgi, Peter T. Hraber, Timothy G. Decker, Andrew G. Smith, Marcos V. Gondim, Lindsey Gillis, Jamie Wandzilak, Gwo-Yu Chuang, Reda Rawi, Fangping Cai, Pierre Pellegrino, Ian Williams, Julie Overbaugh, Feng Gao, Peter D. Kwong, Barton F. Haynes, George M. Shaw, Persephone Borrow, Michael S. Seaman, Beatrice H. Hahn, and Bette Korber

Cell Reports, Volume *25*

Supplemental Information

Completeness of HIV-1 Envelope Glycan Shield

at Transmission Determines Neutralization Breadth

Kshitij Wagh, Edward F. Kreider, Yingying Li, Hannah J. Barbian, Gerald H. Learn, Elena Giorgi, Peter T. Hraber, Timothy G. Decker, Andrew G. Smith, Marcos V. Gondim, Lindsey Gillis, Jamie Wandzilak, Gwo-Yu Chuang, Reda Rawi, Fangping Cai, Pierre Pellegrino, Ian Williams, Julie Overbaugh, Feng Gao, Peter D. Kwong, Barton F. Haynes, George M. Shaw, Persephone Borrow, Michael S. Seaman, Beatrice H. Hahn, and Bette Korber

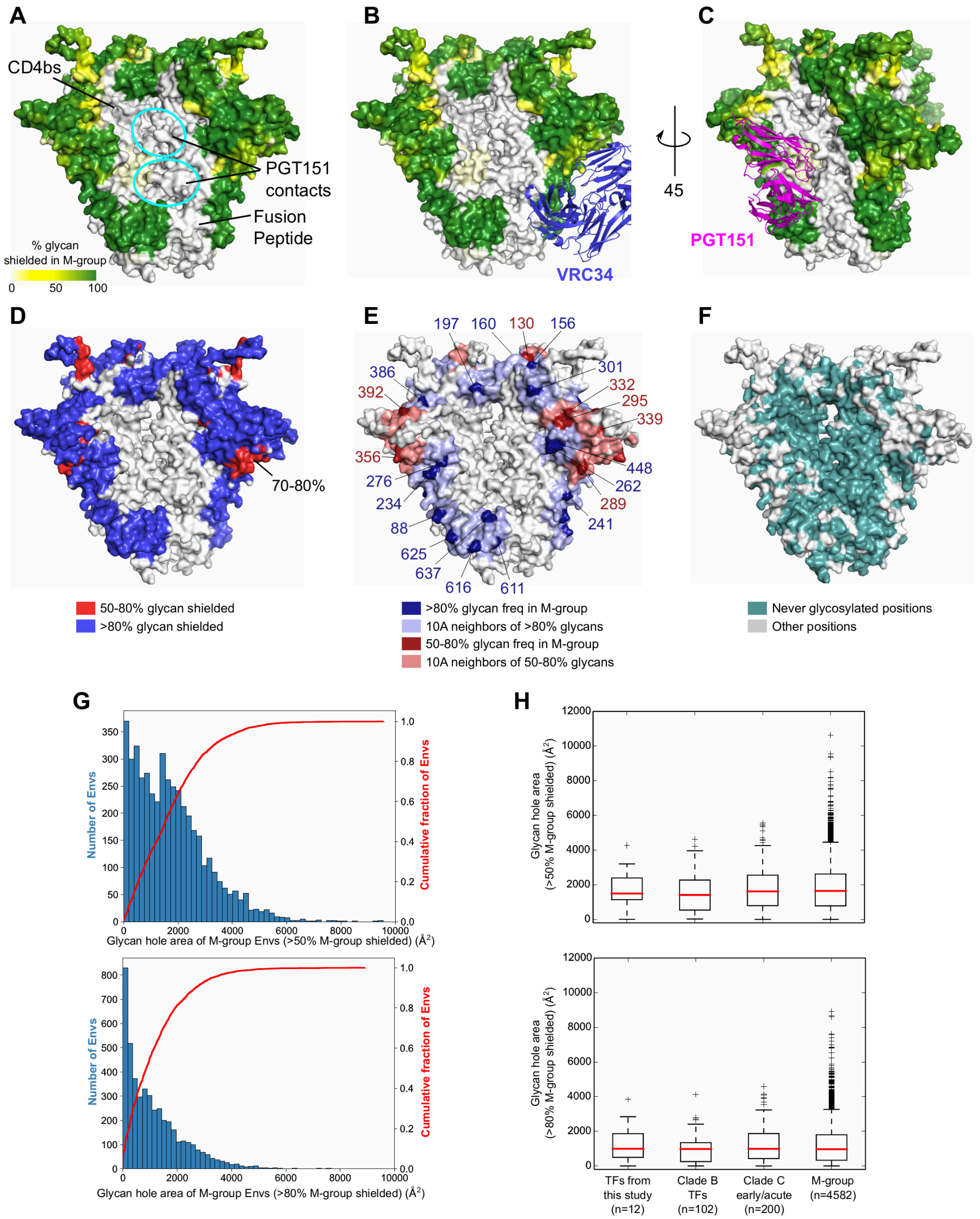

Fig. S1 (related to Figs. 1, 3). Glycan shield in M-group viruses. (A-C) The Env trimer surface is color-coded according the percent of M-group Envs predicted to be glycan shielded. Regions that are never glycan-shielded as well as those that are targeted by broadly neutralizing antibodies are highlighted. The CD4 binding site is well characterized (Zhou et al. 2015). In addition, VRC34 targets the fusion peptide (Kong et al. 2017) and PGT151 targets the gp120-gp41 interface (Lee et al. 2016), both non-glycan-shielded regions. (B) & (C) show models illustrating the approximate interaction of each bNAb with the X1193 crystal structure. For these models, we used bNAb-Env co-crystal structures (PDB: 5I8H and 5FUU) and structurally aligned these to the X1193 structure using the align function in PyMOL. (D) Env

protein surface color-coded using our two cutoffs for M-group conserved glycan shields >80% (blue) and 50-80% (red), repeated from Fig. 1B for context. (E) PNGS frequencies were calculated using 4,582 M-group Envs from the Los Alamos HIV Database “filtered web” alignment and high frequency PNGS are shown in dark blue (>80%) and dark red (50-80%). The 10Å neighbors around each glycan are shown in light blue for >80% PNGS and salmon for 50-80% PNGS. Regions that were within 10Å from both >80% and 50-80% PNGS are colored light blue. (F) Env sites that never had a PNGS motif in 4,582 M-group Envs are highlighted on the trimer structure in teal. These sites are enriched in the regions that our strategy predicts are never glycan shielded in M-group. (G) Frequency distribution (blue bars) and cumulative distribution (red curve) of unshielded areas for 4,582 M-group Envs using either >50% (top) or >80% (bottom) conservation cutoff. (H) Boxplots of glycan hole areas for the 12 TFs from this study, 102 TF subtype B Envs from (Keele et al., 2008), 200 subtype C early/acute Envs from (Rademeyer et al., 2016) and 4,582 database M-group Envs. Top and bottom panels show calculations using >50% and >80% M-group conserved shields, respectively. The distribution of glycan hole areas of the 12 TF Envs from this dataset was similar to those from other groups (p = 0.92-0.96 and 0.69-0.79 using Kolmogorov-Smirnov test for >50% and >80% M-group conserved areas, respectively).

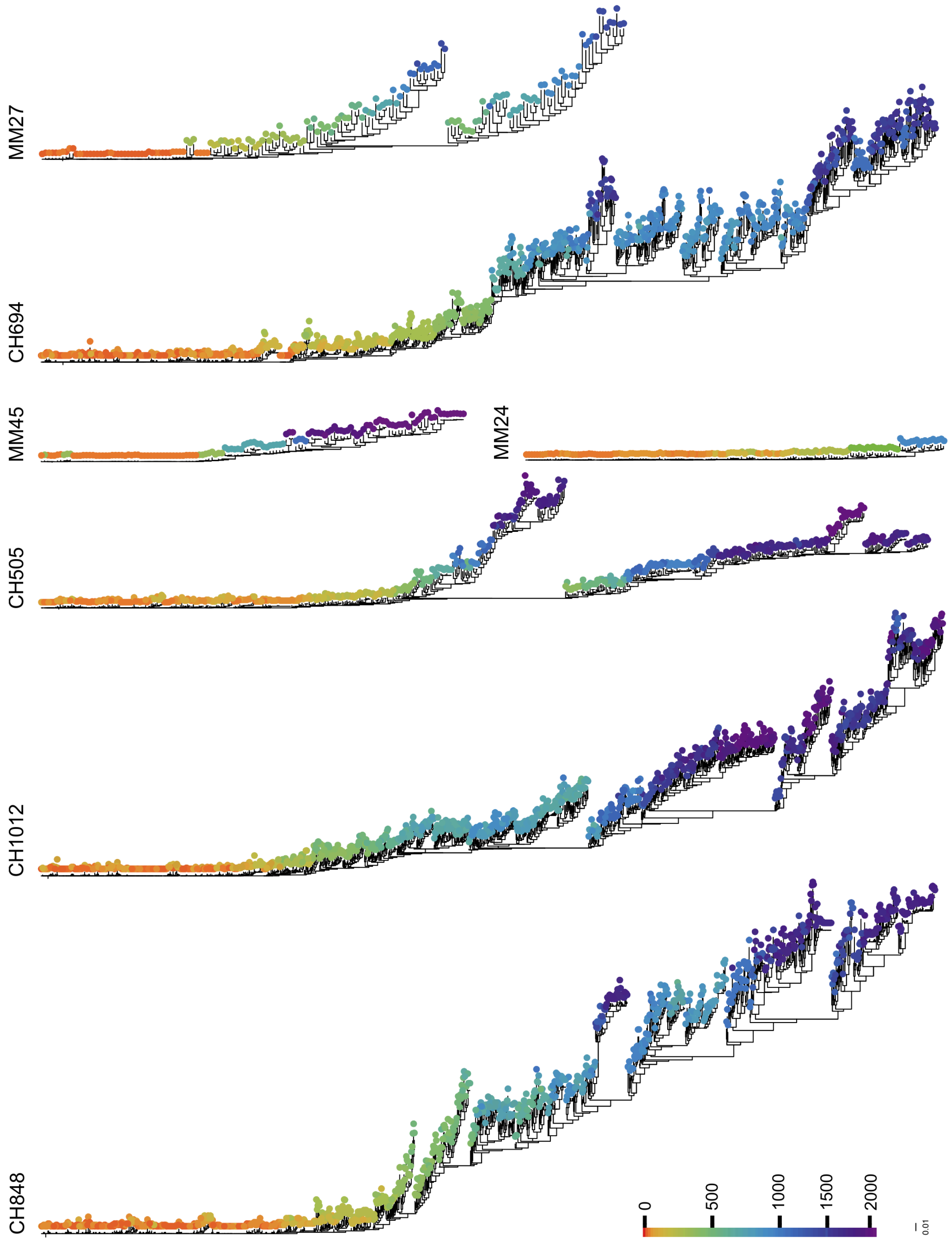

Fig. S2 (related to Figs. 3-7): Maximum-likelihood env phylogenetic trees for subjects who developed high neutralization breadth. The sequences from each subject are shown with filled circles at the leaves of the tree, which are color-coded according to days post infection as shown by the color-bar. Scale bar indicates 0.01 substitutions per site.

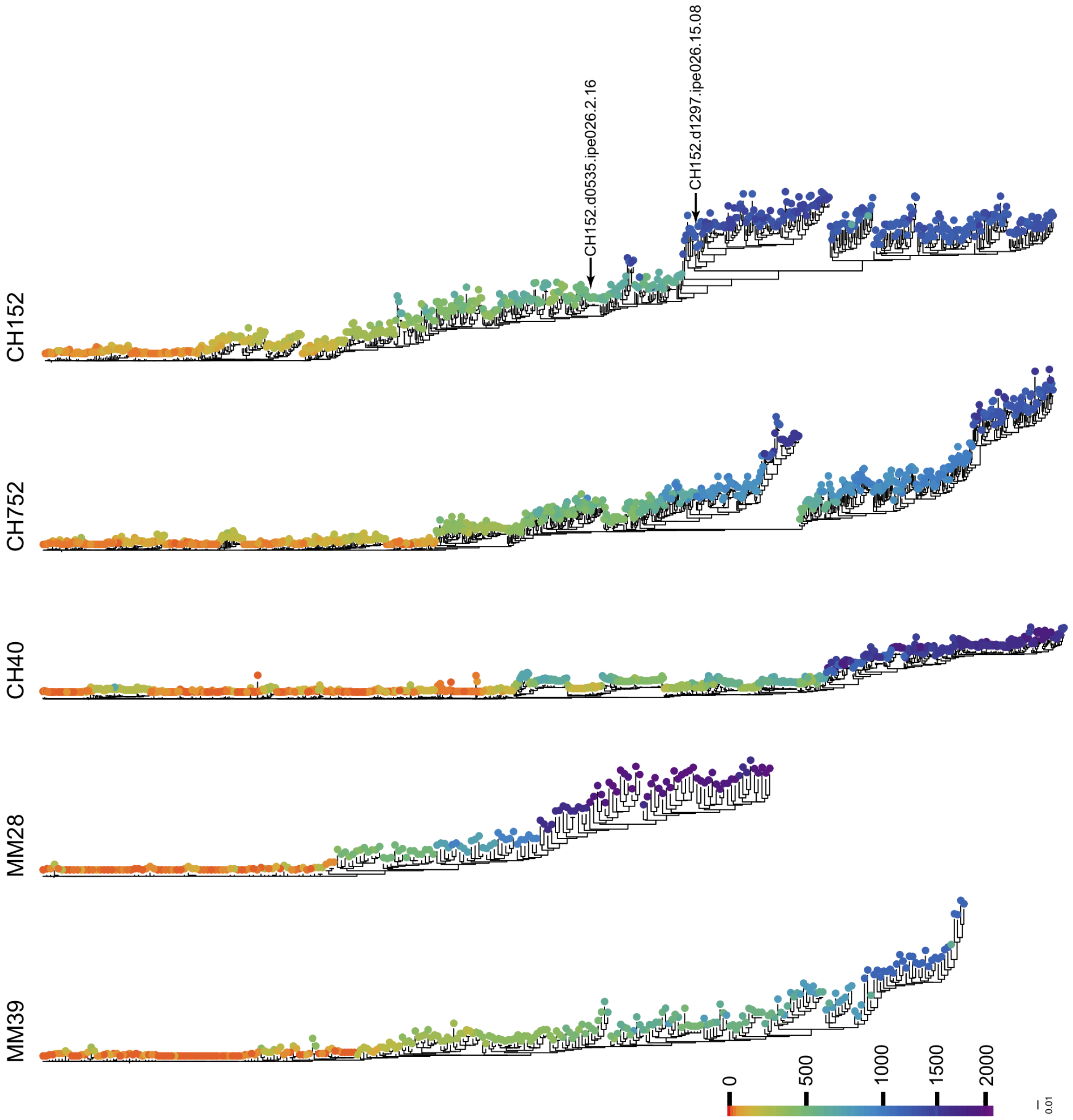

Fig. S3 (related to Figs. 3-7): Maximum-likelihood *env* phylogenetic trees for subjects who developed low neutralization breadth. Same as Fig. S2, but for subjects CH40, CH152, CH752, MM28 and MM39. For CH152, the day 535 and 1297 *envs* used for autologous plasma neutralization (Fig. 4, Table S4) are highlighted.

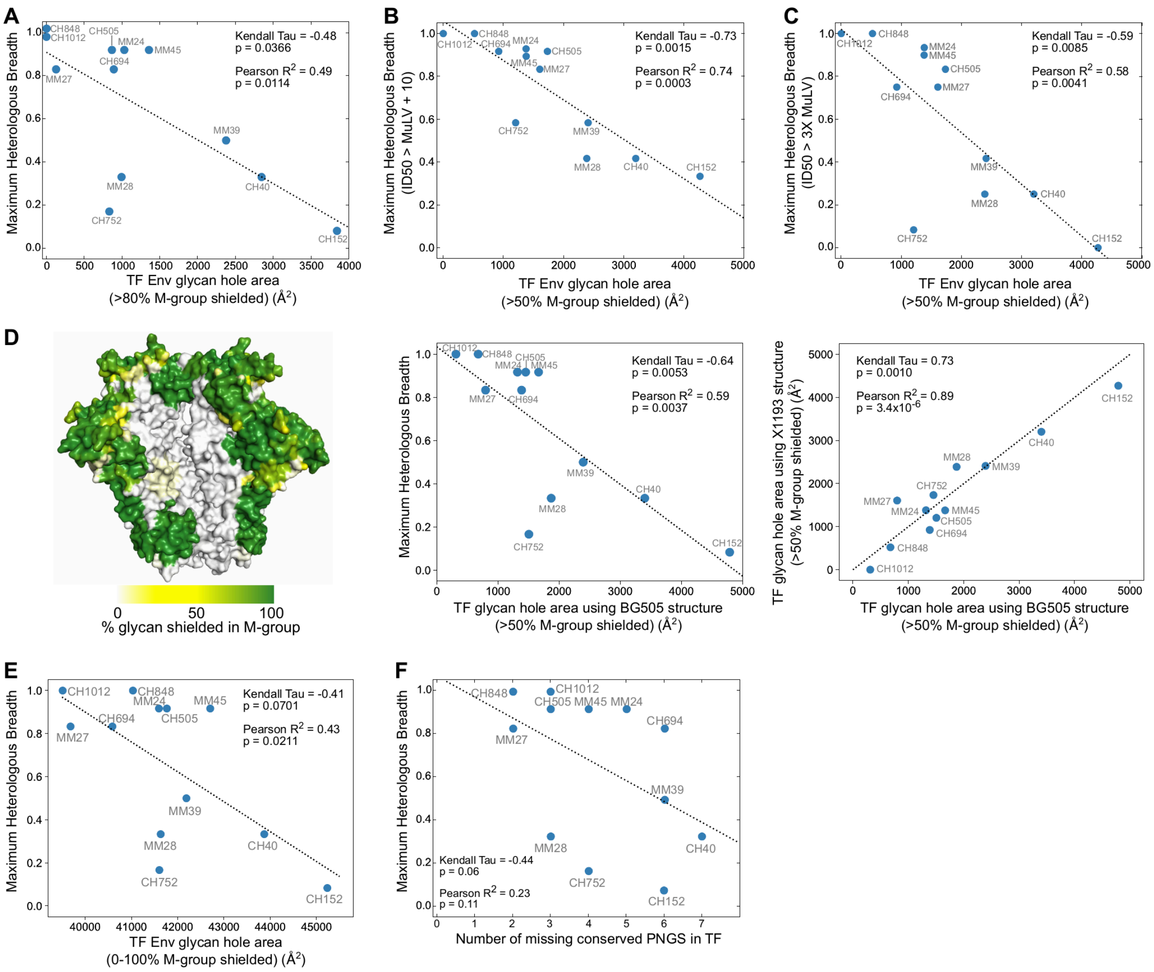

Fig. S4 (related to Fig. 3): (A) Similar to Fig. 3C, except >80% M-group conserved glycan shield was used to calculate the infrequent glycan hole area of TF Envs. (B) Similar to Fig. 3C, except the maximum breadth values were calculated using a different cutoff for detecting positive neutralization, and pseudoviruses for which plasma ID50 titers > MuLV ID50 + 10 were considered as neutralized. (C) Similar to (B), except maximum breadth values calculated using the cutoff of pseudovirus ID50 > 3X MuLV ID50 titer. See Table S3, for plasma ID50 titers and breadth values using different cutoffs. (D) Glycan shield analysis using a subtype A Env structure. Glycan shielding of all 4,582 M-group Envs was recalculated using a subtype A BG505 Env trimer structure (PDB: 5FYL, Stewart-Jones et al., 2016) instead of the reference subtype G X1193 structure for calculation of conservation of M-group glycan shield (left). Similarly, TF glycan shields for the 12 subjects were recalculated using the BG505 structure and compared to the maximum heterologous breadth (middle) and values obtained using X1193 (right). (E) Same as (A), except the full trimer without the hypervariable regions was used for TF glycan hole area calculations. (F) The negative trend between maximum breadth and the number of TF glycan holes as calculated using a sequence-only alignment based approach (see Methods).

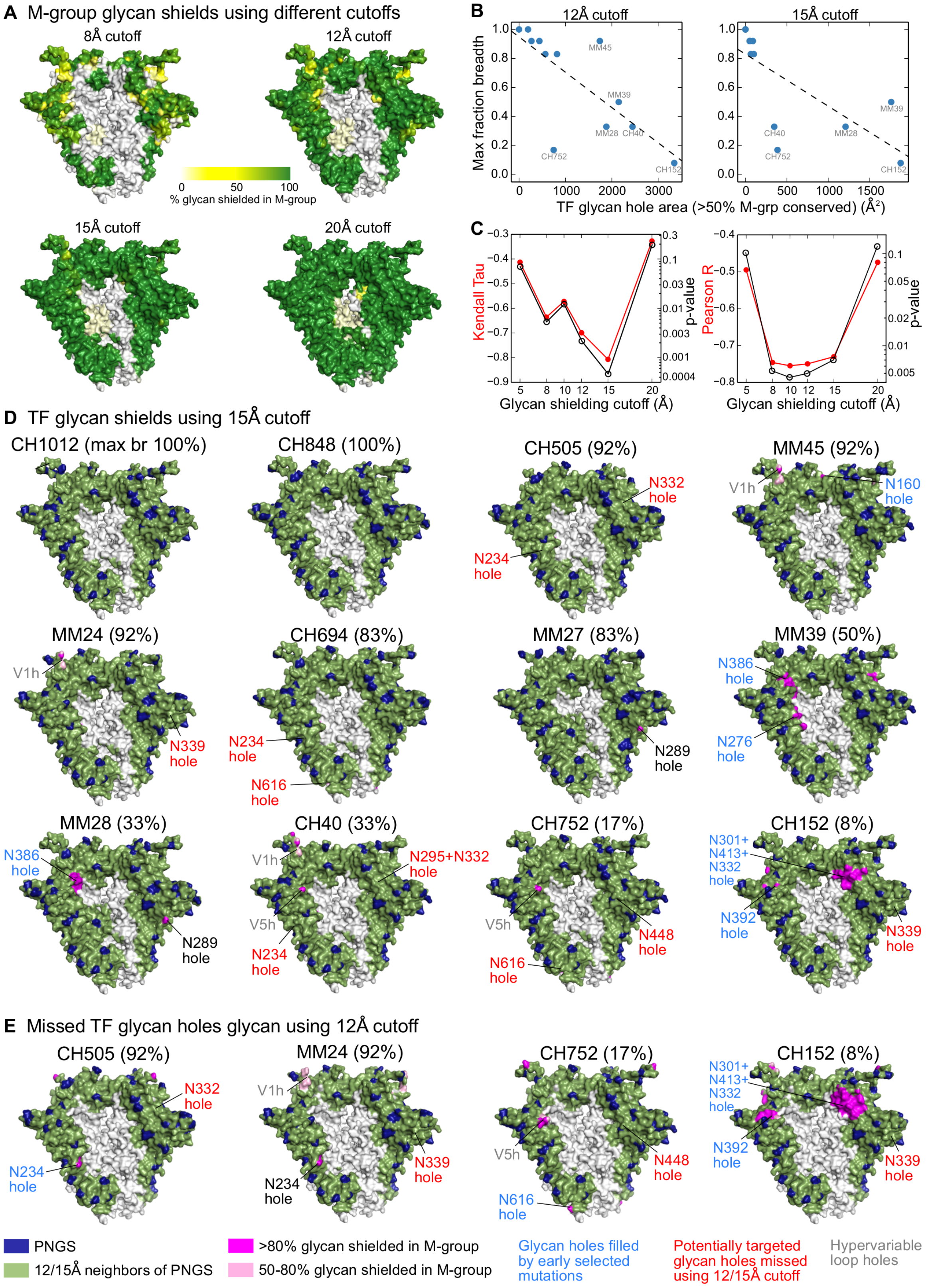

Fig. S5 (related to Figs. 3-5): Glycan shield analyses using different shielding distance cutoffs. We explored the use of different per-glycan shielding distance cutoffs (5, 8, 12, 15 and 20Å), and carried out the same analysis steps as for the 10Å cutoff to calculate the M-group conserved glycan shields and the TF

glycan shields. (A) The M-group conservation of glycan shielding using different cutoffs. (B) For each cutoff, we analyzed the correlation between maximum heterologous breadth and TF glycan hole area using the >50% conserved M-group glycan shield as baseline. The correlations for 12Å and 15Å cutoffs are shown. (C) The statistics of the correlations between maximum heterologous breadth and TF glycan hole area are shown as a function of the cutoff radius using Kendall Tau rank test on the left and Pearson correlation test on the right. In each panel, red curve shows the correlation statistic and black curve shows the p-value. (D) The TF glycan shields are shown using the 15Å cutoff. Glycan holes that are filled by early glycan mutations are indicated in blue, and those that are filled by early glycan hole mutations but are not seen using the 15Å cutoff are indicated by red. (E) Similar to (D) using the 12Å cutoff, and only those TFs for which potentially targeted glycan holes that are missed are shown.

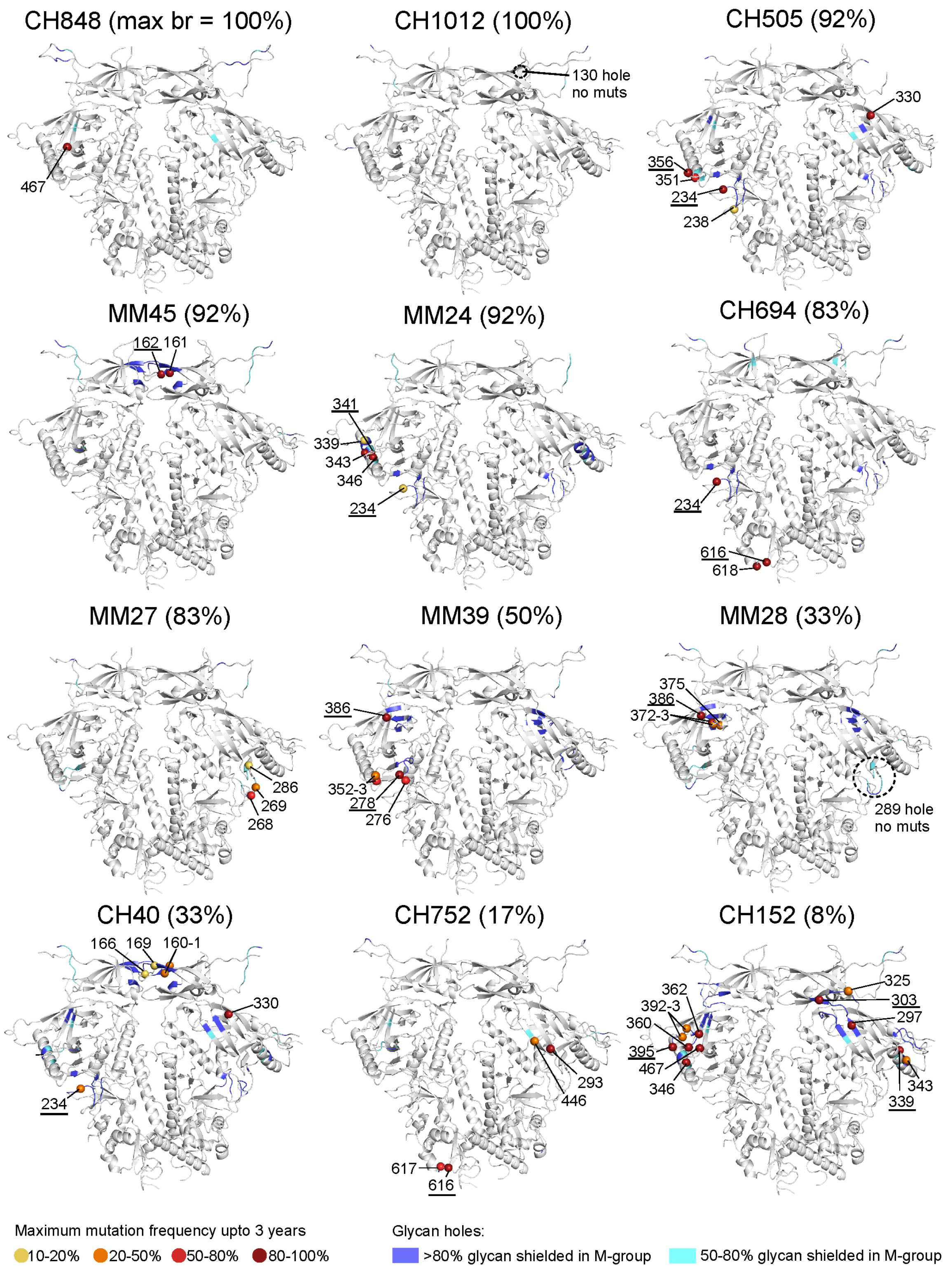

Fig. S6 (related to Figs. 3-6): Early non-synonymous mutations in TF glycan holes. Each panel shows non-synonymous mutated sites that arose in the TF glycan holes in the first 3 years for each subject (Methods). Only those sites that had at least 10% sequences in any time point with mutations away from TF were considered. If the 10% threshold for a time point from any subject was 1 or less, the threshold of at least 2 mutated sequences was used. The mutated sites are shown as beads color-coded according to the

maximum frequency of non-TF sequence (TF loss) up to 3 years post infection. TF glycan holes are shown in blue (>80% M-group glycan shielded) or cyan (50-80% glycan shielded in M-group).

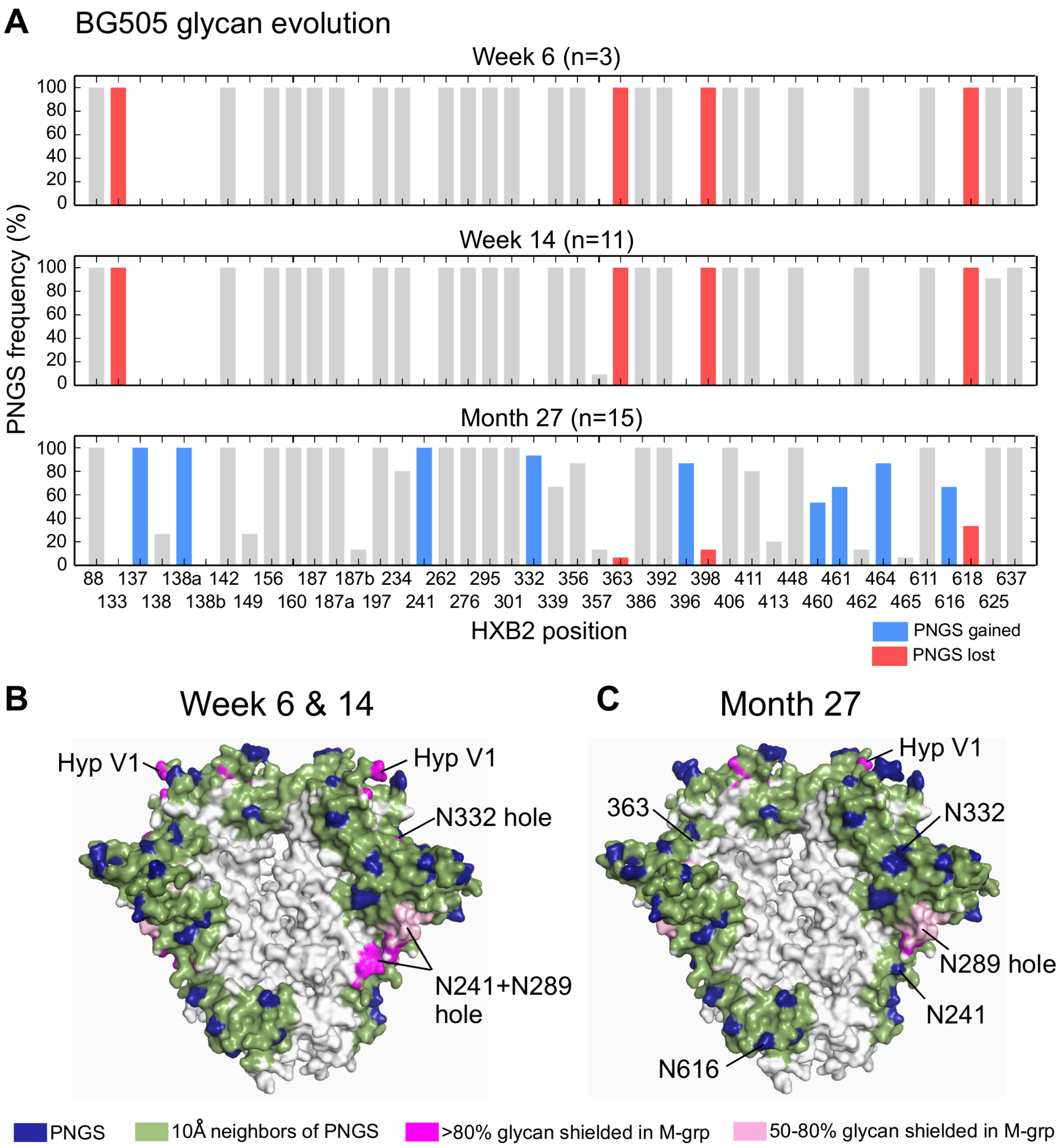

Fig. S7 (related to Figs. 6, 7): Glycan shield evolution in BG505 infant. (A) PNGS frequency for sequences from BG505 infant over time. (B) Consensus glycan shield for week 6 (same as week 14). (C) Consensus glycan shield for month 27.
